# Fine mapping of a male sterility gene *ms*-*3* in a novel cucumber (*Cucumis sativus* L.) mutant

**DOI:** 10.1007/s00122-017-3013-2

**Published:** 2017-11-13

**Authors:** Yike Han, Fengyue Zhao, Shang Gao, Xianyun Wang, Aimin Wei, Zhengwu Chen, Nan Liu, Xueqiang Tong, Xinmeng Fu, Changlong Wen, Zhenxian Zhang, Ningning Wang, Shengli Du

**Affiliations:** 1 Beijing Key Laboratory of Growth and Developmental Regulation for Protected Vegetable Crops, College of Horticulture, China Agricultural University, Beijing, 100193 China; 2 State Key Laboratory of Vegetable Germplasm Innovation, Tianjin Key Laboratory of Vegetable Breeding Enterprise, Tianjin Kernel Cucumber Research Institute, Tianjin, 300192 China; 3 College of Life Sciences, Nankai University, Tianjin, 300071 China; 4 Beijing Vegetable Research Center (BVRC), Beijing Academy of Agricultural and Forestry Sciences, Beijing Key Laboratory of Vegetable Germplasms Improvement, Beijing, 100097 China

## Abstract

*****Key message***:**

**The cucumber male sterility gene** ***ms*** **-** ***3*** **was fine mapped in a 76** **kb region harboring an** ***MMD1*** **-like gene** ***Csa3M006660*** **that may be responsible for the male sterile in cucumber**.

**Abstract:**

A cucumber (*Cucumis sativus* L.) male sterile mutant (*ms*-*3*) in an advanced-generation inbred line was identified, and genetic analysis revealed that the male sterility trait was controlled by a recessive nuclear gene, *ms*-*3*, which was stably inherited. Histological studies suggested that the main cause of the male sterility was defective microsporogenesis, resulting in no tetrad or microspores being formed. Bulked segregant analysis (BSA) and genotyping of an F_2_ population of 2553 individuals were employed used to fine map *ms*-*3*, which was delimited to a 76 Kb region. In this region, a single non-synonymous SNP was found in the *Csa3M006660* gene locus, which was predicted to result in an amino acid change. Quantitative RT-PCR analysis of *Csa3M006660* was consistent with the fact that it plays a role in the early development of cucumber pollen. The protein encoded by *Csa3M006660* is predicted to be homeodomain (PHD) finger protein, and the high degree of sequence conservation with homologs from a range of plant species further suggested the importance of the *ms*-*3* non-synonymous mutation. The data presented here provide support for *Csa3M006660* as the most likely candidate gene for *Ms*-*3*.

**Electronic supplementary material:**

The online version of this article (10.1007/s00122-017-3013-2) contains supplementary material, which is available to authorized users.

## Introduction

In the context of plant breeding, the phenomenon of male sterility represents a powerful means to improve hybrid seed production and to protect the commercial value of parental lines. It can greatly increase the effectiveness of F_1_ hybrid seed production without manual pollination, and reduce the production cost dramatically. In general terms, male sterility can be divided into cytoplasmic male sterility (CMS) and genic male sterility (GMS). CMS, which is maternally inherited, is a result of mitochondrial gene function, and fertility can be restored by nuclear restorer-of-fertility (Rf) genes that can be introduced by crosses with restorer lines (Hanson and Bentolila 2004; Horn 2006). In contrast, GMS is caused by recessive or dominant nuclear genes (Ke et al. 2005). The majority of sterile crop plants have arisen from spontaneous mutants, although a few have been created by mutagen treatments (Kaul 1988; Budar and Pelletier 2001).

In breeding programs, CMS is preferred to GMS, because it allows easier identification of maintainers of male sterility. CMS has been reported in a large number of plant species and has been important in the development of rice (*Oryza sativa*; Virmani 1994), maize (*Zea mays*), and wheat (*Triticum aestivum*) (Budar and Berthomé 2007), cotton (*Gossypium hirsutum*; Subhash 2015), and Brassicaceae crops (Yamagish and Bhat 2014). However, there are some disadvantages to this approach, such as sensitivity to temperature, incomplete sterility, and a limited availability of restorer lines (Fu and Tu 2002). GMS, in contrast, allows stable and complete male sterility and the genes underlying male sterility are relatively easy to transfer. Production of hybrids using GMS has been reported in rice (Kim et al. 2007), maize (Wu et al. 2016), cotton (Subhash 2015), tomato (*Solanum lycopersicum*; Atanassova 2000), and garlic (*Allium sativum*; Mayer et al. 2013), among others. One major drawback of the GMS system is that the progeny of maternal parents segregates as 50% male fertile and 50% male sterile plants, and this requires the eradication of male fertile plants from the maternal line. This problem can be addressed through marker-assisted selection (MAS), which facilitates the identification of different genotypes in segregating generations through the detection of molecular markers (Tanksley et al. 1989). Different types of genetic markers, closely linked to a GMS locus, have been used to identify genotypes in breeding populations, such as sequence tagged site (STS) markers in Chinese cabbage (*Brassica rapa*; Ying et al. 2003), cleavage amplified polymorphic sequence (CAPS) marker in chili pepper (*Capsicum annuum*; Lee et al. 2010), sequence characterized amplified regions (SCAR) and CAPS markers in lettuce (*Lactuca sativa*; Hayashi et al. 2011), and single-nucleotide polymorphism (SNP) markers in cotton (Feng et al. 2015).

Cucumber (*Cucumis sativus* L.), which belongs to the Cucurbitaceae family, is one of the most economically important vegetable crops worldwide (Li et al.^2013^). For the flowers of cucumber which was monoecious and unisexual, male sterility systems can also be widely used in the breeding of cucumber. To date, four main types of male sterility have been reported in cucumber: (1) an apetalous sterile mutant (*ap*); (2) a pleiotropic pollen-aborted mutant (*ms*-*1*); (3) an aborted male flower genotype (*ms*-*2*); and (4) a closed-flower genotype (Qi Zhang et al. 1994). However, these cases of male sterility have not been used in hybrid cucumber seed production; because their inheritance is determined by nuclear genes and they are associated with undesirable traits, such as missing corolla, malformed ovaries, and closed female flowers (Grimbly 1980; Hutchins 1936).

There exists gynoecious sex expression in cucumber both genetically and chemically induction which provides alternative ways for commercial hybrids production. However, most current Chinese cucumber parent lines are genetically monoecious, and it is difficult to achieve 100% female flowers by chemical induction, and additional male flowers’ removal is needed. Gynoecious parental lines cannot be protected, because it can be selfed by chemical male flower induction.

We previously identified a novel male sterile cucumber mutant in the advanced-generation inbred line, YL-5, and here, we report that the inheritance of this male sterile trait is conferred by a single recessive nuclear gene. We designated this male sterile gene *ms*-*3*. Anatomical and histological studies showed that the main reason for the male sterility was the failure of meiosis, leading to no pollen being formed. Based on fine mapping, bulked segregant analysis (BSA), and genotyping of a large F_2_ population using a Kbioscience Allele-specific Polymorphism (KASP) assay, the candidate *ms*-*3* gene, responsible for the male sterility, was identified. Finally, we mapped *ms*-*3* to a 76 Kb genomic DNA region.

## Materials and methods

### Plant material

In June 2012, 36 male sterile cucumber plants that were spontaneous mutation were identified in the Northern Chinese cucumber fertile lines, YL-5, at the experimental farm of Tianjin kernel Cucumber Research Institute in Tianjin, China. Morphological characteristics, including plant height, leaf shape, and fruit traits, were documented. YL-5 male sterile plants were tagged and hand-pollinated with pollen from fertile YL-5 wild-type plants to generate an F_1_ generation. In the autumn of 2012, the F_1_ plants were selfed to generate the F_2_ generation. At the same time, a test crossing was performed, where the YL-5 male sterile plants were hand-pollinated with the heterozygous fertile F_1_ plants, generating C_1_ seeds. The F_2_ seeds and C_1_ seeds were grown in the spring of 2013. Male flower fertility in the F_1_, F_2_, and C_1_ generations was determined. All plants were grown in the greenhouses in Tianjin, China and were under long day-light exposure. The day and night temperature of the greenhouse was controlled at 25–30 and 15–18 °C, respectively.

### Stereo microscopic imaging of stamens

The male flower buds were divided into six stages, according to bud size: stage I, 2–3 mm; stage II, 3–6 mm; stage III, 6–9 mm; stage IV, 9–12 mm; stage V, 12–15 mm; stage VI, > 15 mm. Buds from male sterile and fertile plants were collected around 9:00 am. Corollas were carefully removed with a pair of tweezers and the stamens were then observed and photographed using an Olympus SZX16 stereoscopic microscope (Tokyo, Japan).

### Pollen observation

Sterile and fertile male flowers were collected and placed on wet filter paper in culture dishes, and then kept in an ice box. Anthers were removed and placed on a glass slide, 2–3 drops of distilled water were added, and the anthers were squashed gently with tweezers. Pollen grains were observed and photographed using an Olympus IX70 microscope (Tokyo, Japan).

### Paraffin sectioning and microscopy

Six different stages of buds from male sterile and fertile plants were collected and immersed into fresh FAA (5 mL formalin, 5 mL acetic acid, and 90 mL 70% ethanol) solution, and then kept in an ice box. Paraffin sections were made as previously described (Wang et al. 2013), The slice thickness is 8 µm, using a Leica RM2016 microtome (Leica, Germany).

### Transmission electron microscopy (TEM)

Male flower buds (1–3 mm long) were collected from male sterile and fertile plants and placed in glutaraldehyde. Ultrathin sections were made as previously described (Vignolini et al. 2012), the slice thickness is 70 nm, and TEM images collected using a Hitachi H-7650 transmission electron microscope (Hitachi, Japan).

### Bulked segregant analysis

A cross was made between the YL-5 male sterile mutant (female parent, P_1_) and a genetically distinct male fertile line (D37-1, male parent, P_2_) to create an F_1_ generation. An F_2_ segregating population was then generated by F_1_ self-pollination. Four DNA pools were constructed: the P_1_ pool from the 20 YL-5 mutant (*ms*-*3*/*ms*-*3*) plants; the P_2_ pool from the 20 D37-1 (*Ms*-*3/Ms*-*3*) plants; the male fertile (MF) pool from the 50 male fertile plants of the F_2_ generation (*Ms*-*3*/*Ms*-*3* and *Ms*-*3*/*ms*-*3*); and the male sterile (MS) pool from the 50 male sterile plants the of F_2_ generation (*ms*-*3*/*ms*-*3*). These DNA pools were sequenced using the Illumina HiSeq 2500 platform with paired-end reads of 100 bp (Illumina, USA).

### Sequencing data analysis

The raw reads from the four DNA pools were filtered and aligned to the cucumber genome sequence (Chinese long; v2) using the Burrows–Wheeler alignment tool (BWA) (Huang et al. 2009; Langmead and Salzberg 2012). GATK software was used to detect single-nucleotide polymorphisms (SNPs) and InDels (McKenna et al. 2010). The SNP-index and the Δ(SNP-index) (DePristo et al. 2011) values were calculated to identify candidate genomic regions associated with male sterility. The Δ(SNP-index) was determined based on the difference in the SNP-index between the MF and MS pools. An average of Δ(SNP-index) of SNPs located in the given genomic interval was calculated using sliding window approach with 1 Mb window size and 10 kb increment. The Δ(SNP-index) of MF and MS pools and their corresponding SNP-index within the specified window size were plotted in a graph to generate SNP-index plots. The Δ(SNP-index) value should be significantly different from 0 if a genomic region harboring the target gene (Takagi et al. 2013). We calculated statistical confidence intervals of Δ(SNP-index) for all the SNP positions with given read depths and 99% confidence intervals were obtained. By examining the Δ(SNP-index), the plot peak regions above the confidence value were defined as candidate regions for association with male sterility.

### SNP primer design and KASP genotyping

We employed KASP genotyping platform at the Beijing Vegetable Research Center (Beijing, China) to identify the SNP genotype in the segregation population and to construct a genetic map. According to the principle that the base type of the P_1_ pool was the same as the MS pool and the P_2_ pool was the same as the MF pool, all the SNP loci obtained from the sequencing data were filtered for primers design. For each SNP, two forward DNA primers were designed that matched the SNP allele. FAM and VIC fluorophores were used to label the 5′ of the primers to distinguish between the different SNP genotypes, and two forward primers were used with one reverse primer (Table S1). SNP genotyping was conducted following the manufacturer’s protocol (LGC genomics, UK). The “touch-down” PCR program for KASP SNP genotyping was: 95 °C for 15 min, 10 cycles at 94 °C for 20 s; 61 °C (− 0.6 °C/cycle) for 1 min; followed by 26 cycles of 94 °C for 20 s and 55 °C for 60 s.

### Mapping population

KASP was performed to identify the SNP genotypes in 2553 individuals from the F_2_ population of the above-mentioned YL-5 mutant and D37-1 plants to construct a genetic map. Genomic DNA was extracted from young leaves using the CTAB method (Murray and Thompson 1980). To construct the genetic map, the KASP genotyping data were analyzed using the JoinMap 4.0 software (Stam 1993) with default parameters and an LOD score of 10 (Van Ooijen 2006).

### Sequence analysis and prediction of the candidate gene

The sequence and gene functional annotations were obtained from the Cucurbit Genomics Database (http://www.icugi.org/cgi-bin/ICuGI/index.cgi). Conserved domains were identified in the NCBI (https://www.ncbi.nlm.nih.gov/) and SMART (http://smart.embl-heidelberg.de/) databases. Amino acid sequences were aligned using DNAMAN7.0 (Lynnon Biosoft, Quebec, Canada) and ClustalX2.0 (Larkin et al. 2007) software packages.

### Extraction of nucleic acids and qRT-PCR

Total RNA was extracted using a MiniBEST Universal RNA Extraction kit (TaKaRa, Japan) and the first-strand cDNA was prepared using a PrimeScript RT Reagent Kit (TaKaRa, Japan). Quantitative RT-PCR was conducted using an SYBR Premix Ex Taq II kit (TaKaRa, Japan). The candidate gene primers for qRT-PCR were designed using Beacon Designer 7.9 software (PREMIER, PaloAlto, CA, USA) (forward primer: 5′AATCTCCTCCTCCGTGTAG3′; reverse primer: 5′CTGCGTATTCTAACCGTCTC3′) and the cucumber Ubiquitin extension protein (UBI-ep) gene was used as the reference gene (forward primer: 5′CACCAAGCCCAAGAAGATC3′; reverse primer: 5′TAAACCTAATCACCACCAGC3′). Three biological and technical replicates were used for qRT-PCR. Average relative expression levels for each sample were calculated.

### Phylogenetic analysis

Phylogenetic tree was constructed using the MEGA 7 software with a bootstrap method and 1000 replications (Kumar et al. 2016).

## Results

### Genetic analysis of male sterile lines

The male sterile mutant was characterized as having a normal corolla in both male and female flowers, normal fertility in the females, but an absence of pollen from male flowers that otherwise appeared normal. In segregation groups, all the F_1_ plants were fertile, whereas their F_2_ plants exhibited a segregation of 948 male fertile and 321 male sterile plants, giving a ratio close to 3:1. C_1_ plants from the test crossing exhibited a segregation of 103 male fertile and 104 male sterile plants, effectively corresponding to 1:1 ratio. The results above suggested that the male sterility trait was controlled by a pair of nuclear recessive genes.

### Observation of stamens and pollens

**Fig. 1:**
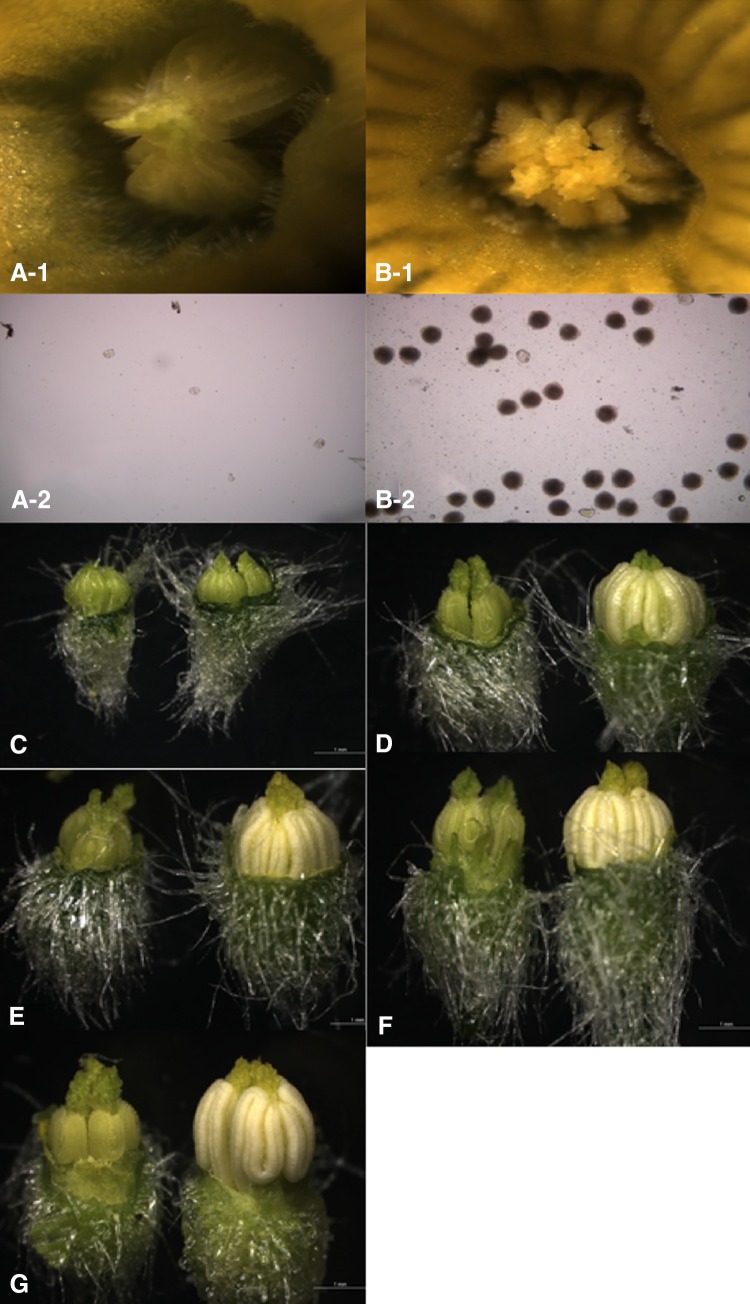
Flowers of male sterile and fertile cucumber plants. **a-1** Sterile stamen and anther, ×80; **a-2** male sterile pollen, ×200; **b-1** fertile stamen and anther, ×80; **b-2** fertile pollen, ×200; **c** comparison of sterile stamen (left) and fertile stamen (right) in stage I, ×20; **d** comparison of sterile stamen (left) and fertile stamen (right) in stage II, ×20; **e** comparison of sterile stamen (left) and fertile stamen (right) in stage III, ×20; **f** comparison of sterile stamen (left) and fertile stamen (right) in stage IV, ×20; **g** comparison of sterile stamen (left) and fertile stamen (right) in stage V, ×20

**Fig. 2:**
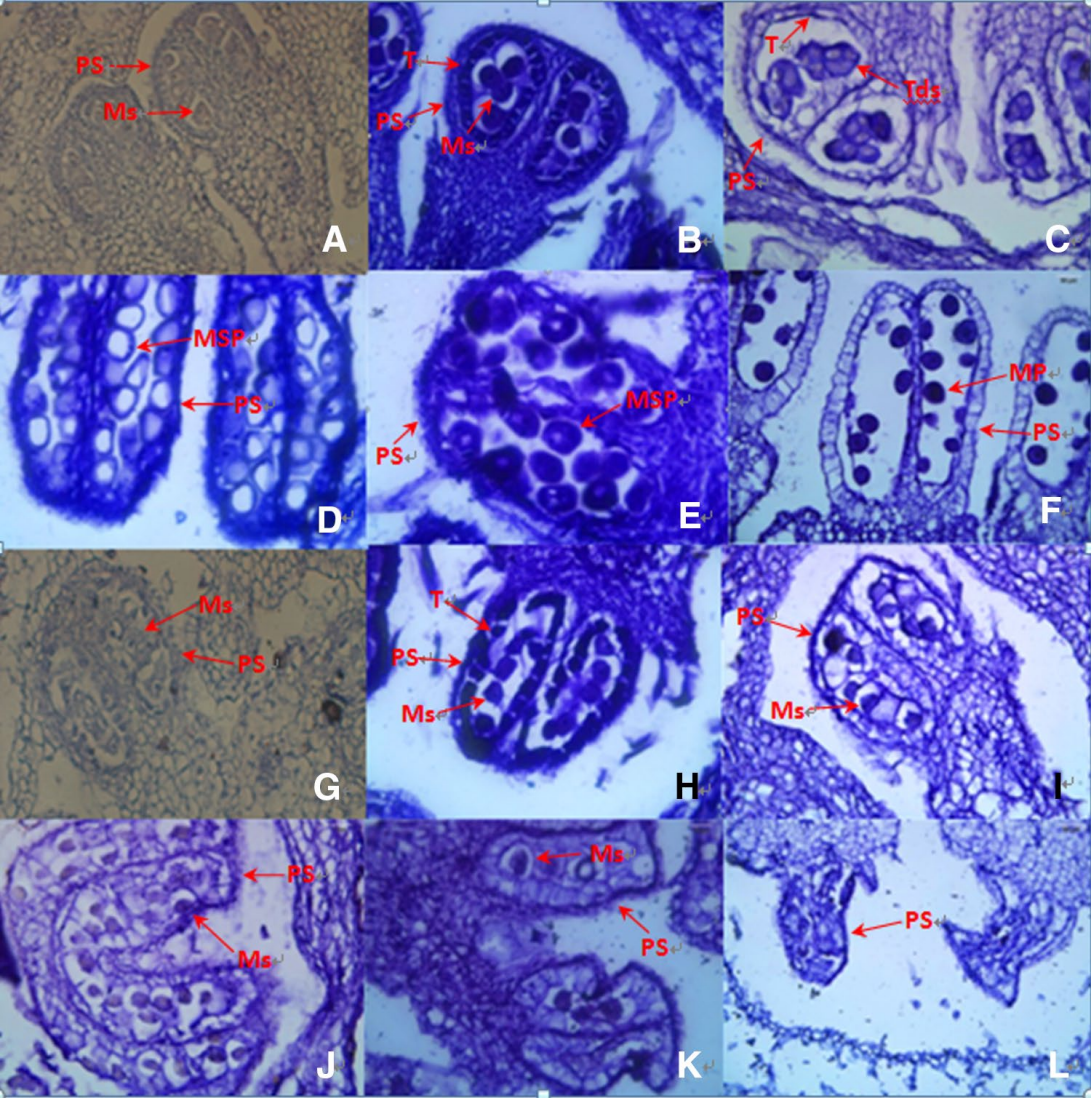
Microstructural analysis of microspore development in cucumber male sterile and fertile lines. **a**–**f** Microspore development in male fertile plants; **g**–**l** microspore development in male sterile plants. *PS* pollen sac, *T* tapetum, *Ms* microsporocyte, *Tds* tetrads, *Msp* microspore, *MP* mature pollen; ×500

Male flower buds at different stages were carefully dissected and observed by stereo microscopy. In the flowering stage, stamens from the male sterile plants were smaller, shriveled, transparent, and pale green. No pollen grains were observed on the anther surface and on the glass slide (Fig. 1a-1, a-2). In contrast, anthers from the male fertile plants were larger, more plump, opaque, and pale yellow (Fig. 1b-1). Many pollen grains were observed on the surface of the anthers and on the glass slide (Fig. 1b-1, b-2).

At stage I, no significant difference was found in stamens from male sterile and fertile plants; however, from stages II to V, significant differences were observed. Anthers from the male sterile plants were smaller, whereas anthers from the fertile plants were larger and plump (Fig. 1c–g).

### Microspore development

Paraffin embedded sections of anthers were observed using differential interference contrast microscopy (Fig. 2). In stage I, normal pollen sacs were observed in the fertile flowers (Fig. 2a), whereas in the male sterile flowers, pollen sacs were irregular (Fig. 2g). At stage II, the microsporocytes, the tapetal cells, and pollen sac cells were normal and well structured (Fig. 2b). In contrast, in the male sterile flowers, obvious morphological abnormalities, including irregularly shaped pollen sacs, microsporocytes and tapetal cells were observed (Fig. 2h). In stage III (Fig. 2c), normal tetrads were observed in the male fertile anthers, whereas no tetrads were observed in the male sterile anthers. The microsporocytes and pollen sacs were morphologically abnormal (Fig. 2i). From stage IV to stage VI, the tetrads developed microspores and matured pollens in fertile anthers (Fig. 2d–f). In contrast, no tetrads or microspores and pollens were observed in the male sterile plants, the tapetal cells and microsporocytes disintegrated and disappeared, and the pollen sacs were malformed and, eventually completely atrophic (Fig. 2j–l). These cytological observations revealed the obvious differences between fertile and sterile plants, and that the microsporocytes failed to produce microspores in the male sterile plants. The results further indicated that the onset of cucumber male sterility occurred in stage I.

Male flowers of 1, 2, and 3 mm in length were sectioned and observed by TEM (Figure S1). In bud length of 1 mm, normal sporogenic cells with visible nucleus were observed in both fertile and sterile flowers, and that the degree of cytoplasmic staining was similar. (Figure S1A, S1D). In bud length of 2 mm, the sporogenic cells developed to microsporocytes, and that the area around the nucleus was distinct, with larger nucleoli and smaller vacuoles in fertile flowers. In contrast, the area around the nucleus was indistinct, with smaller nucleoli and larger vacuoles in microsporocytes of sterile plants (Figure S1B, S1E). In bud length of 3 mm, the fertile microsporocytes developed well and were larger. In contrast, the sterile microsporocytes were seriously vacuolated and smaller (Figure S1C, S1F). The results indicated that the sporogenic cells failed to develop normal microsporocytes in the male sterile plants.

### BSA and genetic mapping

**Fig. 3:**
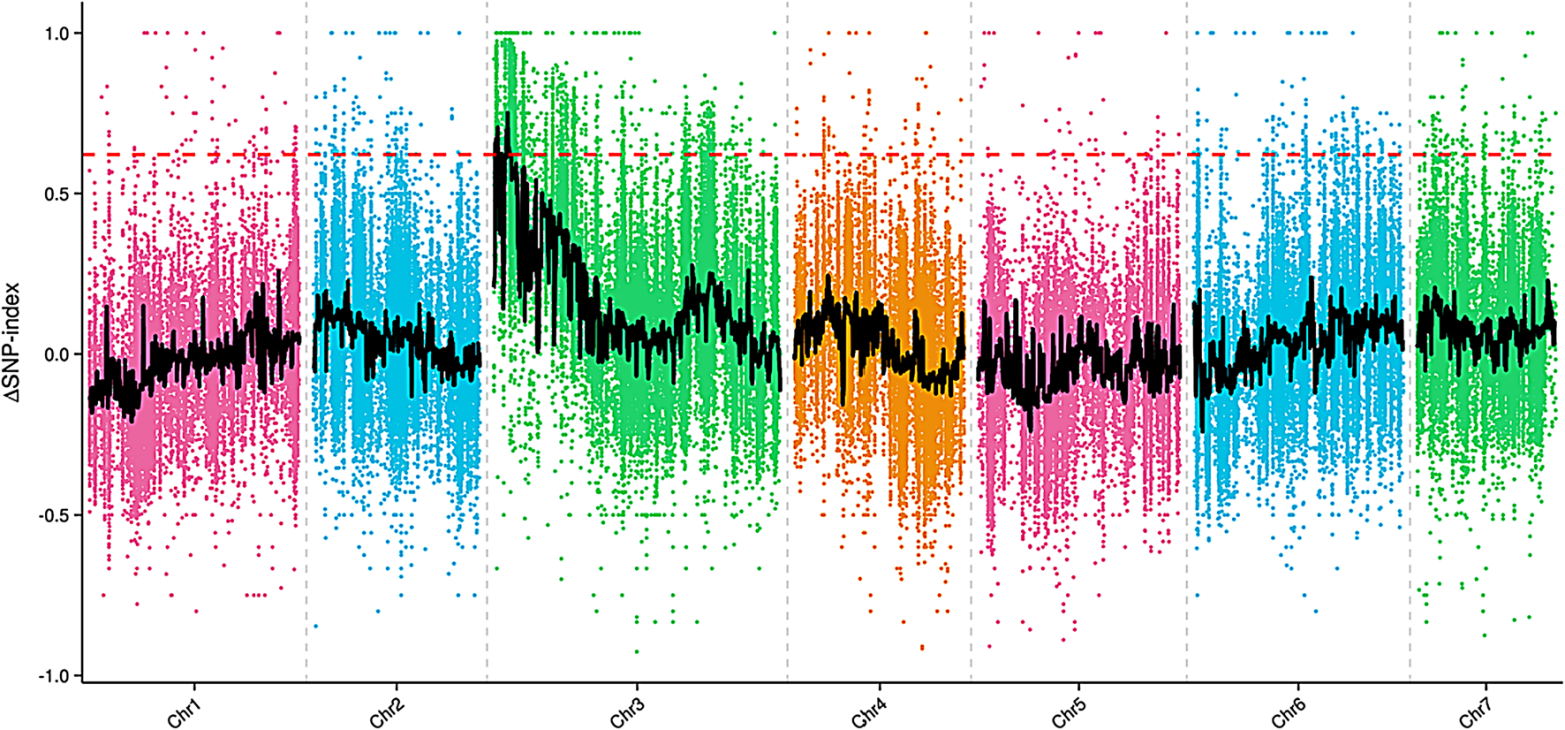
Δ(SNP-index) graph of MF (male fertile) and MS (male sterile) pool from the bulked segregant analysis (BSA). The X-axis represents the chromosome position. The Y-axis represents the Δ(SNP-index) value. The spots represent Δ(SNP-index) values calculated by the formula: [Δ(SNP-index) = SNP-index_Largest-SNP-index_Smallest]. The black line corresponds to the fitted value of the Δ(SNP-index) and the red line represents the confidence value (99%). Two candidate regions (region 1 166,710–564,531, size 397 Kb; and region 2 1,954,776–2,371,279, size 416 Kb) above the confidence value were identified on cucumber chromosome 3

After resequencing, a total of 38.5 Gb were generated, with an average genome sequencing depth of 19× and a coverage of 98.75%. The sequence reads of the four DNA pools were aligned to the cucumber reference genome (cucumber Chinese long genome v2), and many SNPs loci and InDels were found. A total of 42,167 SNPs were found between male fertile (MF) and male sterile (MS) pool. The Δ(SNP-index) was calculated based on the SNP-index of the male fertile (MF) and male sterile (MS) pool. Δ(SNP-index) graphs were generated and the red line in Fig. 3 shows the confidence value (99%). The Δ(SNP-index) value should be significantly different from 0 if a genomic region harboring the target gene. At 99% significance level, only two regions on the end of chromosome 3 were significantly different from 0, which spanned a total of 813 Kb (region 1 166,710–564,531, size 397 Kb; region 2: 1,954,776–2,371,279, size 416 Kb). These results indicated that *ms*-*3* was located at the two candidate regions.

**Fig. 4:**
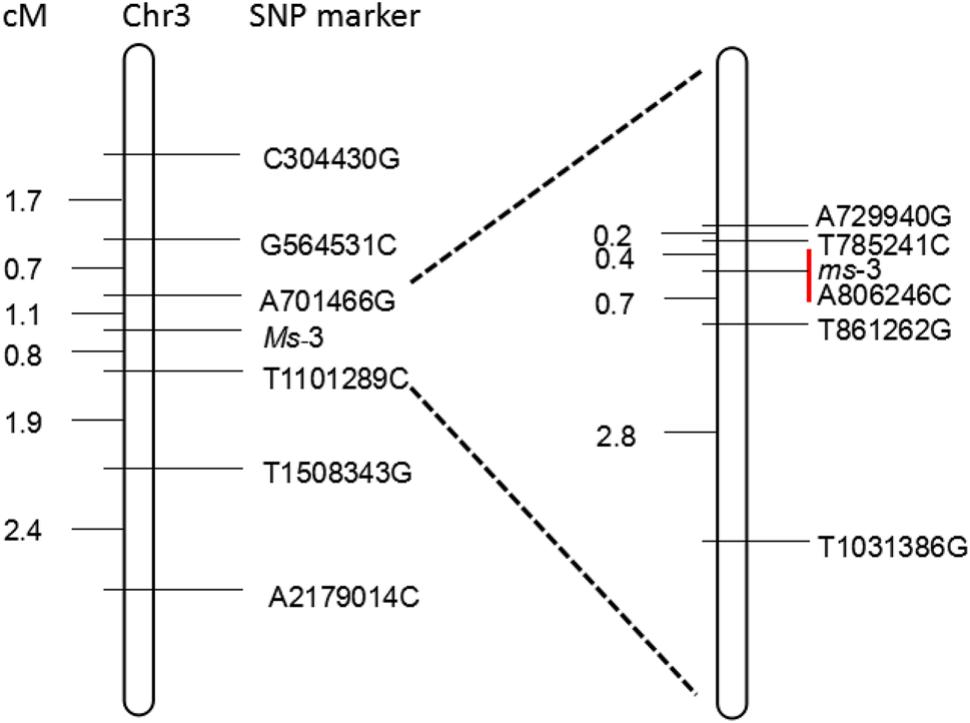
Fine mapping of the cucumber *ms*-3 gene. Single-nucleotide polymorphism (SNP) markers were selected from the region (166,710–2,371,279), and genotyping in an F2 population of 2553 individuals delimited *ms*-3 to a 76 Kb region with flanking marker T785241C and T861262G, respectively, with two and four recombinants. One non-synonymous mutation was detected in *Csa3M006660* between WT and ms-3

6 SNP markers were selected from the region 166,710–2,371,279 and tested for linkage to *ms*-*3* in 938 individuals of the F_2_ population, based on the sequence data. Two SNP markers, A701466G (with four recombinants) and T1101289C (with eight recombinants), were found to be the closest flanking markers for *ms*-*3*. Next, 5 SNP markers between A701466G and T1101289C were selected for fine mapping of the *ms*-*3* in 2553 F_2_ population and the closest flanking markers for *ms*-*3* were found to be T785241C (with 2 recombinants) and T861262G (with 4 recombinants) (Fig. 4). T785241C and T861262G were 1.1 cM apart, corresponding to physical distance of 76 Kb, based on the cucumber genome sequence (Chinese long; v2), and these were located in the middle of the two candidate regions. SNP marker information is provided in Supplementary Table S1.

Using the cucumber genome database as a reference, 11 genes were predicted to be located between T785241C and T861262G. The positions and putative functions of these predicted genes are shown in Supplementary Table S2. Three SNPs and one InDel were existed between T785241C and T861262G between male fertile (MF) and male sterile (MS) pool, however, only non-synonymous SNP was detected in *Csa3M006660* between WT (YL-5 wild type) and *ms*-*3* (YL-5 mutant type). Taken together, our results suggested that *Csa3M006660* was the candidate *Ms*-*3* gene.

### Candidate gene screening and expressing analysis

We cloned *Csa3M006660* from both WT and *ms*-*3*. The gene annotation suggested the presence of 3 exons and 2 introns (Supplementary Figure S2) and the predicted coding sequence (CDS) of the cloned *Csa3M006660* cDNA was 1995 bp, with a predicted corresponding protein length of 664 amino acids. Alignment of the *Csa3M006660* gene sequence between the wild type and the mutant revealed a single non-synonymous T → G mutation in the third exon, resulting in the conversion of a tyrosine (Tyr) to an aspartic acid (Asp) at residue 420. According to the Cucurbit Genomics Database, *Csa3M006660* was predicted to encode a homeodomain (PHD) finger protein that is typically characterized as Cys4HisCys3.

To investigate the conservation of the male sterile mutant locus, 28 genetically distinct fertile cucumber inbred lines were selected and their *Csa3M006660* sequences were determined. All fertile lines shared the WT sequence in this locus (Supplementary Figure S3).

**Fig. 5:**
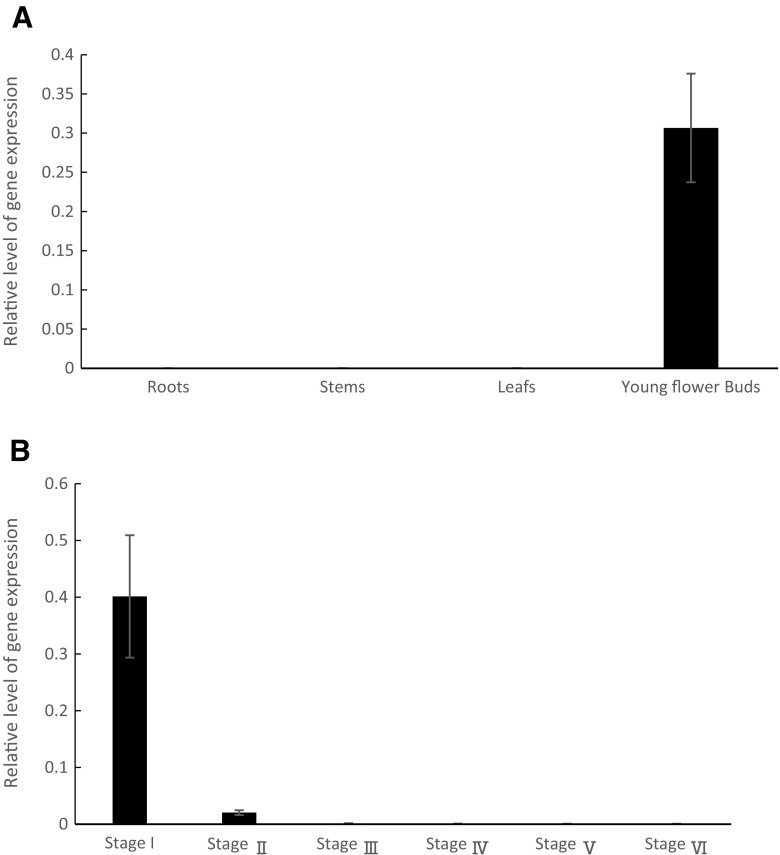
Expression analysis of the *Csa3M006660* gene in cucumber organs. *Csa3M006660* expression in **a** roots, stems, leaves, and young flower buds of wild type (WT); and **b** different stages of flower bud development: stage I (shorter than 3 mm), stage II (3–6 mm), stage III (6–9 mm), stage IV (9–12 mm), stage V (12–15 mm), and stage VI (longer than 15 mm). Expression levels were determined using q-PCR, and values were normalized using UBI-ep as the reference gene. Error bars indicate standard deviation from three biological replicates

The expression of *Csa3M006660* was investigated in WT roots, stems, leaves, and young flower buds and we observed that it was only expressed in young flower buds (Fig. 5a). We then investigated expression levels in the different stages of flower bud development and showed that *Csa3M006660* expression in stage I (bud length 2 3 mm) was significantly higher than in the other stages (Fig. 5b).

### Phylogenic analysis

**Fig. 6:**
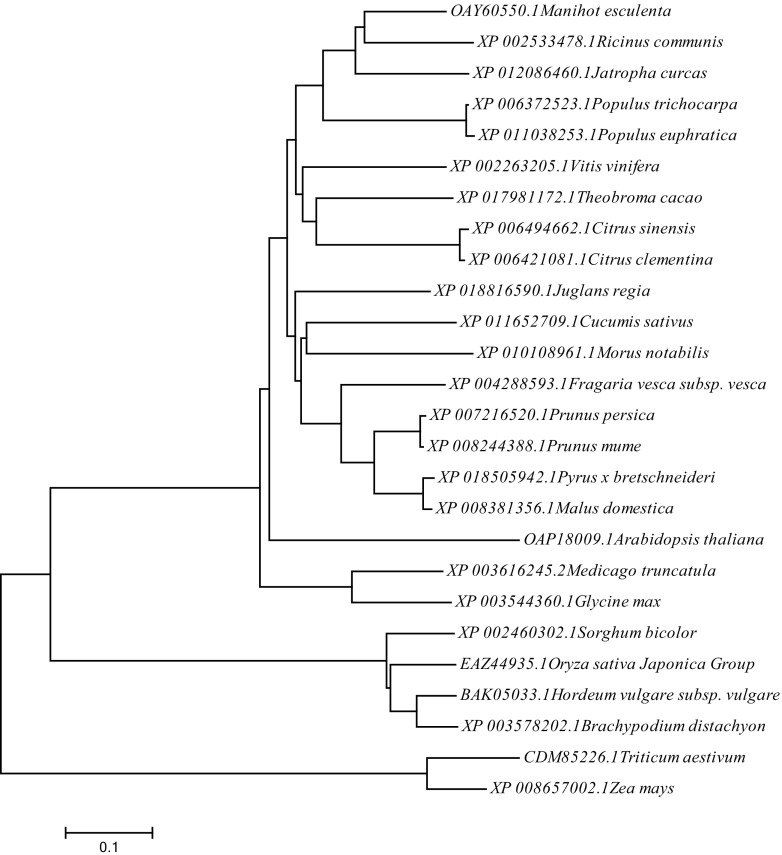
Phylogenetic analysis of Ms-3 and its homologs in 25 other plant species. Evolutionary relationships were inferred using the neighbor-joining method. The ID numbers refer to the gene IDs in the NCBI database and the names of the species are given. The bootstrap test values (1000 replicates) are shown next to the branches. The tree is drawn to scale, with branch lengths in the same units as those of the evolutionary distances used to infer the phylogenetic relationships

To better understand the relationship between Csa3M006660 protein and its close homologs, we searched public databases NCBI using BLAST with the Csa3M006660 amino acid sequence and used an alignment of the closest homologs from 17 additional species and 8 commercial crops for a phylogenetic analysis (Fig. 6). The resulting neighbor-joining tree showed that Csa3M006660 from cucumber grouped together with homologs from *Morus notabilis*. We found that the site where the non-synonymous mutation occurred was highly conserved and that all the proteins had a typical PHD domain, which is a Cys4HisCys3 protein conversed motif (Supplementary Figure S4), including some commercial crops, such as *Hordeum vulgare*, *Triticum aestivum,* and so on.

The results of the alignment revealed that the Csa3M006660 protein shared 48.96% sequence identity with *Arabidopsis thaliana* MMD1, and both shared the same PHD domain and nucleotide in the site where the non-synonymous mutation was located (Supplementary Figure S5). These data presented indicate that *Csa3M006660* is the most likely candidate gene for *Ms*-*3* and that a non-synonymous SNP is responsible for the male sterile phenotype.

## Discussion

### Abnormal meiosis is the cause of male sterility in *ms*-*3*

According to pollen abortion variants and the main features of male sterility, its underlying causes can be classified into different types, including: abnormal meiosis (Nonomura et al. 2004; Xiao 2016); abnormal callose metabolism (Ostergaard et al. 2002); early or late degeneration of tapetum cells (Li et al. 2006); critical chemical changes in pollen wall development (Jung et al. 2006); failure in anther dehiscence (Steiner-Lange et al. 2003), and other types (Kaneko et al. 2004). In this study, the male sterile sporogenous cells appeared to be normal and could generate middle layer cells, tapetal cells, and microsporocytes. However, the microsporocytes were highly vacuolated, with fewer cytoplasmic ribosomes and plastids, and could not produce tetrads and microspores. Thus, the cucumber male sterile *ms*-*3* mutant belongs to the type of male sterility caused by abnormal meiosis. Two types of pollen abortion male sterility have been reported: (1) a pleiotropic pollen-aborted mutant (*ms*-*1*), whose most distinguishing external characteristic was the failure of anthesis in the staminate flowers. The pollen sterility varied from 30 to 90%, and was found to be controlled by a pair of recessive genes (Shifriss 1950); and (2) male sterile-2 (*ms*-*2*), whose staminate flower buds usually aborted when they were approximately three quarters of the way through development, and where pollen abortion did not occur until after the mitotic division of the pollen grain nucleus (Barnes 1960). The *ms*-*2* mutation was found to be in a single recessive gene (Whelan 1972). In contrast, the cucumber male sterile mutant described in this current study is completely pollen sterile, and the onset of sterility occurs early in the pollen mother cell, which is different from the above-mentioned *ms*-*1* and *ms*-*2* mutants. We designated the male sterile gene as *ms*-*3.*

In *A. thaliana* and rice, anther development has been well studied and many of the genes involved in meiosis have been identified. For example, rice *PAIR1*, *PAIR2,* and *PAIR3*, and *A. thaliana SYN1*/*DIF1* are important for chromosome condensation and pairing (Nonomura et al. 2004, 2006; Yuan et al. 2009; Bai et al. 1999). *A. thaliana SWITCH1*/*DYAD* and rice *REC8* are essential for sister chromatid cohesion and bivalent formation (Mercier et al. 2003; Shao et al. 2011), and *A. thaliana ASK1*, *ATK1,* and *MPS1* play important roles in spindle formation and chromosome segregation (Yang et al. 1999; Chen et al. 2002; Jiang et al. 2009). In addition, *A. thaliana MMD1*/*DUET* was shown to be involved in regulating meiotic cell cycle progression (Yang et al. 2003). Mutations in these genes also cause defective meiocyte development and male sterility.

### *Csa3M006660* is the most likely candidate gene underlying the *ms*-*3* mutation

Through BSA and genotyping, we identified the *ms*-*3* gene in a 76 Kb candidate region at the end of chromosome 3, which contained 11 predicted genes. In *Csa3M006660*, a single non-synonymous mutation was detected in the third exon. Further sequence alignment and expression analysis suggested that the non-synonymous mutation site of *Csa3M006660* is highly conserved and that the protein may play an important role in the early development of cucumber pollen. Thus, we propose that *Csa3M006660* is the candidate gene underlying the male sterility phenotype.

Amino acid alignments suggested that the Csa3M006660 protein shares 48.96% sequence identity with *A. thaliana* MMD1, and that the proteins have the same PHD domain and amino acid in the site of the non-synonymous *ms*-*3* mutation.

In *A. thaliana*, MMD1 (also known as DUTE) encodes a PHD finger protein required for normal male meiosis (Reddy et al. 2003), and an *mmd1* mutation was shown to cause abnormal cell death of male meiocytes (Yang et al. 2003). MMD1 plays a role in meiotic I-independent cell division by regulating the expression of two genes related to meiosis, *TDM* and *JAS*, which have critical functions in cell cycle transition and spindle organization (Andreuzza et al. 2015). The PHD finger can interact with different modified histone peptides and is required for specific binding to H3K3me2 (Patel and Wang 2013). A recent study showed that MMD1 is required for progressive compaction of prophase I chromosome to metaphase I bivalents, by ensuring the progression of male meiotic chromosome condensation, a process in which the PHD domain is essential (Wang et al. 2016). MMD1 regulates the expression of the condensin gene, *CAP*-*D3*, by directly binding to its promoter region in male meiocytes (Wang et al. 2016). Our analyses suggested that Csa3M006660 and MMD1 may have similar functions.

Taken together, the data presented above provide support for *Csa3M006660* as the most likely candidate gene for *Ms*-*3*. However, more evidence is needed to definitively demonstrate this.

### Application of the novel male sterile mutant in cucumber breeding

The traditional breeding system using GMS is very laborious and time-consuming as visible characteristics linked to male sterility have not been found so far and the phenotype is recognized only after flowering. To breed GMS cucumber maternal parent lines, consecutive backcrosses are performed to introduce the recessive *ms*-*3* gene. MAS has been utilized to select the GMS individuals. Based on our result, the SNP marker A806246C can be used to detect the presence of *ms*-*3* gene by KASP genotyping, which makes it easier and more efficient for the selection of *ms*-*3* individuals in the breeding population.

In GMS system, the progeny, which from the cross of homozygous recessive male sterile and heterozygous male fertile plant, will segregate 50% male fertile and 50% male sterile. This problem precludes the application of GMS system in hybrid seed production. An effective approach to overcome the problem has been reported in maize using the Seed Production Technology (SPT) (Wu et al. 2016). An SPT construct was employed in this technology, which contains a complementary wild-type male fertility gene, a pollination disruption gene, and a seed screenable marker gene. A heterozygous SPT maintainer line could be generated by transformed the SPT construct into a homozygous recessive male sterile line. The SPT maintainer could only produce pollen grains without SPT transgenes as expression of the pollination disruption gene blocks the pollen germination. Hence, it is capable of propagating non-transgenic and homozygous nuclear male sterile lines for use as female parents in hybrid production. In cucumber, we can also construct an SPT maintainer through transgenic method as in maize. Crossing of SPT maintainer with the male sterile plants will produce homozygous male sterile lines for hybrid production. We infer that the SPT process may be the most effective, practical, and safe method to utilize the cucumber *ms*-*3* gene for hybrid seed production.

#### **Author contribution statement**

YH, FZ, SG, NW, and SD designed the experiments. YH, XT, XF, and NL conducted genetic analysis and developed the F_2_ populations. FZ, SG, and XW analyzed the sequencing data. FZ performed genetic and physical mapping, and conducted gene expression analysis and phylogenic analysis. SG, ZC, and AW conducted observation of stamens, pollens, and microspore development. CW performed SNP marker development and KASP genotyping. YH, FZ, SG, ZZ, and SD wrote the paper. NW and SD supervised the experiments. All authors read and approved the final manuscript.

## Electronic supplementary material

Below is the link to the electronic supplementary material.

Supplementary material 1 (PDF 83 kb)

Supplementary material 2 (PDF 69 kb)

Supplementary material 3 (PDF 68 kb)

Supplementary material 4 (PDF 105 kb)

Supplementary material 5 (PDF 77 kb)

Supplementary material 6 (PDF 49 kb)

Supplementary material 7 (PDF 42 kb)
