# Supplementary material for: Fine mapping of a male sterility gene ms-3 in a novel cucumber (Cucumis sativus L.) mutant
Source: Theor Appl Genet. 2017 Nov 13;131(2):449–60. doi: 10.1007/s00122-017-3013-2 (PMC5787221; doi:10.1007/s00122-017-3013-2)
Supplement: Supplementary file 1 — Supplementary material 1 (PDF 83 kb) [file 122_2017_3013_MOESM1_ESM.pdf]

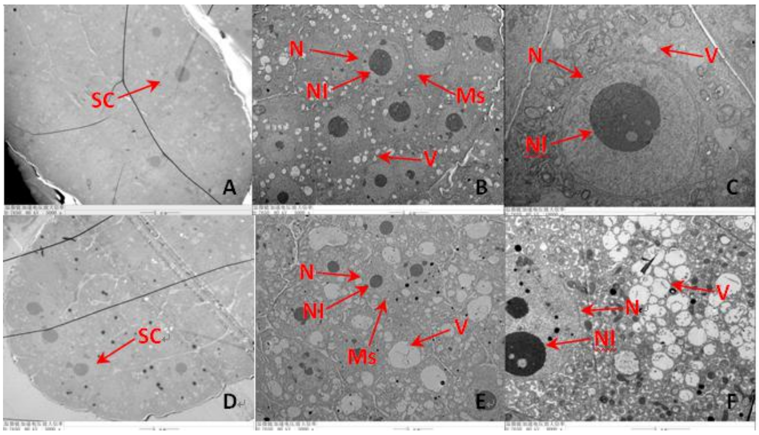

Fig_S1. Transmission electron microscopy (TEM) images of male sterile and fertile anthers. (A) Sporogenous cells of a male fertile anther, ×5000; (B) microsporocyte of a male fertile anther at an early stage, ×5000; (C) microsporocyte of male fertile anther, ×10000; (D) Sporogenous cells of a sterile anther, ×5000; (E) microsporocyte of a sterile anther at an early stage, ×5000; (F) microsporocyte of a sterile anther, ×8000. SC: sporogenous cell; N: nucleus; Nl: nucleoli; V: vacuoles; Ms, microsporocyte.
