# Supplementary material for: Fine mapping of a male sterility gene ms-3 in a novel cucumber (Cucumis sativus L.) mutant
Source: Theor Appl Genet. 2017 Nov 13;131(2):449–60. doi: 10.1007/s00122-017-3013-2 (PMC5787221; doi:10.1007/s00122-017-3013-2)
Supplement: Supplementary file 2 — Supplementary material 2 (PDF 69 kb) [file 122_2017_3013_MOESM2_ESM.pdf]

Fig S2. Alignment of *Csa3M006660* sequences from WT and *ms-3*
(Yellow highlight=exons; Red highlight=SNP)

```
WT. seq      CAAAACCTCCAAATCCAAATATGAAACTACCAAAATTCAGTGAATGGTAGAGAAAGAAGA
ms-3. seq    CAAAACCTCCAAATCCAAATATGAAACTACCAAAATTCAGTGAATGGTAGAGAAAGAAGA
             ************************************************************

WT. seq      AGAAGAAGAAGATGAAGAAATGAAGTTCATCTTCAGCCATGTCGATTTCGATTCTGGAAT
ms-3. seq    AGAAGAAGAAGATGAAGAAATGAAGTTCATCTTCAGCCATGTCGATTTCGATTCTGGAAT
             ************************************************************

WT. seq      CCTGCAAGAAGAGAAAAAGAAGGCCTAAACTTTTCGGGTTTCAAACGTTCGGGGATCCTG
ms-3. seq    CCTGCAAGAAGAGAAAAAGAAGGCCTAAACTTTTCGGGTTTCAAACGTTCGGGGATCCTG
             ************************************************************

WT. seq      GATCGCCAATCAACCCCACGGGTCCATTTCGTGAGAATATCAGAATCTTTCTTCAACAAT
ms-3. seq    GATCGCCAATCAACCCCACGGGTCCATTTCGTGAGAATATCAGAATCTTTCTTCAACAAT
             ************************************************************

WT. seq      GTGCAGAGATTGAAGATTACAGAATTCAAGAAATGCCTATATGGTGTACTCTCCTTGTTC
ms-3. seq    GTGCAGAGATTGAAGATTACAGAATTCAAGAAATGCCTATATGGTGTACTCTCCTTGTTC
             ************************************************************

WT. seq      ATGAAAATAAAAGCTTCGTTGTTCCACTTTACACTATTGAAGAAGATGTGAAGCTCTCCC
ms-3. seq    ATGAAAATAAAAGCTTCGTTGTTCCACTTTACACTATTGAAGAAGATGTGAAGCTCTCCC
             ************************************************************

WT. seq      CAAAACCCTACTGCGATCAATGCCGATGTTCTGGTTTGAATTCTTTTTCTCTACTGAGTT
ms-3. seq    CAAAACCCTACTGCGATCAATGCCGATGTTCTGGTTTGAATTCTTTTTCTCTACTGAGTT
             ************************************************************

WT. seq      TCTCTGTTTCTATGGATTCTGTTCATGGCTGATTTGTTTTTTCTGATTTTTTTTTCAGGG
ms-3. seq    TCTCTGTTTCTATGGATTCTGTTCATGGCTGATTTGTTTTTTCTGATTTTTTTTTCAGGG
             ************************************************************

WT. seq      TGGAGTAATCATTTTGTATCGAAAAGAAAATATCATATCGTAATACCGTTGGATGATCGG
ms-3. seq    TGGAGTAATCATTTTGTATCGAAAAGAAAATATCATATCGTAATACCGTTGGATGATCGG
             ************************************************************

WT. seq      TGGAACAAACGATTAGACGATGGCGGTTTCGACCTCGATGATCAAACTCATCTTCTTCAT
ms-3. seq    TGGAACAAACGATTAGACGATGGCGGTTTCGACCTCGATGATCAAACTCATCTTCTTCAT
             ************************************************************

WT. seq      GGATTGATTCACTGCAATGGCTTCGGGCATTTGCTCTGCGTCAATGGAATCGAAGGAGGA
```

```
ms-3.seq     GGATTGATTCACTGCAATGGCTTCGGGCATTTGCTCTGCGTCAATGGAATCGAAGGAGGA
             ************************************************************

WT.seq       TCCAAGTTTCTTTGTGGCAGAGAAGTTATGGATCTTTGGGATAGAATCTGCACAAATCTA
ms-3.seq     TCCAAGTTTCTTTGTGGCAGAGAAGTTATGGATCTTTGGGATAGAATCTGCACAAATCTA
             ************************************************************

WT.seq       AGAACAAGGTGTAAAGAATGGAAGTTATGAATGACTATGATAAAATTGTTGTTCTTCTCT
ms-3.seq     AGAACAAGGTGTAAAGAATGGAAGTTATGAATGACTATGATAAAATTGTTGTTCTTCTCT
             ************************************************************

WT.seq       GCCGCAAAAGAAATTTTCTCTAACTTTCTGTTTGGTTCTTAGGAAAATTACAGTTGAGGA
ms-3.seq     GCCGCAAAAGAAATTTTCTCTAACTTTCTGTTTGGTTCTTAGGAAAATTACAGTTGAGGA
             ************************************************************

WT.seq       TTTATCCAAGAAACGATCAATGGATCTACGTCTTCTTCATGGGGTAGCATACGGTCATCC
ms-3.seq     TTTATCCAAGAAACGATCAATGGATCTACGTCTTCTTCATGGGGTAGCATACGGTCATCC
             ************************************************************

WT.seq       ATGGTTTGGGAGATGGGGCTACAGATTTTGCCGAGGAAGCTTTGGAGTGAAAGAACACCA
ms-3.seq     ATGGTTTGGGAGATGGGGCTACAGATTTTGCCGAGGAAGCTTTGGAGTGAAAGAACACCA
             ************************************************************

WT.seq       TTACAGTAGAGCTTTGGAAATCCTCAGCTCTCTGGAACTCGACAAGATAATGCACGAAGT
ms-3.seq     TTACAGTAGAGCTTTGGAAATCCTCAGCTCTCTGGAACTCGACAAGATAATGCACGAAGT
             ************************************************************

WT.seq       CGACTATAGCGATCGAGGAAGAGAAGTGAAGCAAATCATTCGACATTATCGAAATCTGAG
ms-3.seq     CGACTATAGCGATCGAGGAAGAGAAGTGAAGCAAATCATTCGACATTATCGAAATCTGAG
             ************************************************************

WT.seq       TGAAACGCAGTTGATCACACTGAAAGATCTACTGAAGTTCATGTTGACAGTAAAATATGT
ms-3.seq     TGAAACGCAGTTGATCACACTGAAAGATCTACTGAAGTTCATGTTGACAGTAAAATATGT
             ************************************************************

WT.seq       TTCTGCGATCGAGAAGAAAACGGTTCAACCAATCGCTAAATCTCCTCCTCCGTGTAGACA
ms-3.seq     TTCTGCGATCGAGAAGAAAACGGTTCAACCAATCGCTAAATCTCCTCCTCCGTGTAGACA
             ************************************************************

WT.seq       ATCTCTGCAGCGAAACAAGCAGCAATCTCTAGTGAAGGAGAAGCAAATACGGTACAGAAA
ms-3.seq     ATCTCTGCAGCGAAACAAGCAGCAATCTCTAGTGAAGGAGAAGCAAATACGGTACAGAAA
             ************************************************************

WT.seq       ATTCGCCACTGCAATTTCTAATATGGACAGCCGATGGCCGGCGAGACGGTTAGAATACGC
```

ms-3.seq ATTCGCCACTGCAATTTCTAATATGGACAGCCGATGGCCGGCGAGACGGTTAGAATACGC
****

WT.seq AGCGGAGGTGATTGTGAAAGCATTGGAAGAGAAGAAATCAGATAAATTCAGCCATGGCGG
ms-3.seq AGCGGAGGTGATTGTGAAAGCATTGGAAGAGAAGAAATCAGATAAATTCAGCCATGGCGG
****

WT.seq AAATGGAATGACTCGTCAAGATGTTCGAGATGCTGCTCGCCTTCACATCGGCGACACTGG
ms-3.seq AAATGGAATGACTCGTCAAGATGTTCGAGATGCTGCTCGCCTTCACATCGGCGACACTGG
****

WT.seq ATTGCTCGATTACGTTCTAAAATCACTGAACAACGTGATCGTAGGTAACCAAATAGTTCG
ms-3.seq ATTGCTCGATTACGTTCTAAAATCACTGAACAACGTGATCGTAGGTAACCAAATAGTTCG
****

WT.seq CCGTGCAGTGAATCCTAAAACACGAATTTTAGAGTACACGATTCATGAACTTAGAAATGG
ms-3.seq CCGTGCAGTGAATCCTAAAACACGAATTTTAGAGGACACGATTCATGAACTTAGAAATGG
********************************** *************************

WT.seq CATTCAATTAACAGAAGAGCAAGAATCAACAGAAAATTCAGAACCAACCGTAACTCCTGG
ms-3.seq CATTCAATTAACAGAAGAGCAAGAATCAACAGAAAATTCAGAACCAACCGTAACTCCTGG
****

WT.seq CAAAGACATTTACAACGACGTGCTATGTATATACAGAAGCATTTTCCTTGACTATCCAGA
ms-3.seq CAAAGACATTTACAACGACGTGCTATGTATATACAGAAGCATTTTCCTTGACTATCCAGA
****

WT.seq ATCAGAAATGGTAGAATTAGCAACCCAGGGAGTTCTCGATAGTAAACATTTTGCTAAAGA
ms-3.seq ATCAGAAATGGTAGAATTAGCAACCCAGGGAGTTCTCGATAGTAAACATTTTGCTAAAGA
****

WT.seq ATGGCCTCTTCAAGATGAAGAAGAGCATCTATTGACGTTCATTATCAAATTGATGCCGAG
ms-3.seq ATGGCCTCTTCAAGATGAAGAAGAGCATCTATTGACGTTCATTATCAAATTGATGCCGAG
****

WT.seq GCTAACTTTTACACATACGGATTTAGAGTTGAAGAGTGATTTCATGCCATCCGGCGAGGT
ms-3.seq GCTAACTTTTACACATACGGATTTAGAGTTGAAGAGTGATTTCATGCCATCCGGCGAGGT
****

WT.seq AGTGGTTCTTCCACTACACACAACAATCGGAGAAGTAAAAGAAGCAGCAGAAAAAGCTCT
ms-3.seq AGTGGTTCTTCCACTACACACAACAATCGGAGAAGTAAAAGAAGCAGCAGAAAAAGCTCT
****

WT.seq AAGAGACACATATTACGTTACGGAACAGTTCGAGGTTTTGGCGATAGAGAATTTGGAGAA

```
ms-3.seq    AAGAGACACATATTACGTTACGGAACAGTTCGAGGTTTTGGCGATAGAGAATTTGGAGAA
            ************************************************************

WT.seq      TTACGAAGACAGAGAGGTGATTTTTGGAGCGGTAGAATCGGGAGCAGAGTTGTTTGTGAA
ms-3.seq    TTACGAAGACAGAGAGGTGATTTTTGGAGCGGTAGAATCGGGAGCAGAGTTGTTTGTGAA
            ************************************************************

WT.seq      AGGAATGGGTATTGATTTAGATACACCATTGAAGTACCAAGGAGGAGTTGGTACATGGAA
ms-3.seq    AGGAATGGGTATTGATTTAGATACACCATTGAAGTACCAAGGAGGAGTTGGTACATGGAA
            ************************************************************

WT.seq      AGTTCGATGTGAGTGTGGCACCGGAGACGACGATGGCGAGAGGATGGTGGCTTGTGACAT
ms-3.seq    AGTTCGATGTGAGTGTGGCACCGGAGACGACGATGGCGAGAGGATGGTGGCTTGTGACAT
            ************************************************************

WT.seq      ATGCGAGATCTGGCAGCACACTCGCTGCTGTGGAATCGACGACGCTGATAATGTGCCGTT
ms-3.seq    ATGCGAGATCTGGCAGCACACTCGCTGCTGTGGAATCGACGACGCTGATAATGTGCCGTT
            ************************************************************

WT.seq      GTTGTTCGTATGCGCCGCCTGCTGCGACTCGCTTGGACAATTAAAAATTTGAATGGAATT
ms-3.seq    GTTGTTCGTATGCGCCGCCTGCTGCGACTCGCTTGGACAATTAAAAATTTGAATGGAATT
            ************************************************************

WT.seq      TTGTTTGTATGCGCCGTTGTTCGGGTGATGACTTCATTGACTAAACTTTTTGTTTGTAAT
ms-3.seq    TTGTTTGTATGCGCCGTTGTTCGGGTGATGACTTCATTGACTAAACTTTTTGTTTGTAAT
            ************************************************************

WT.seq      TACATTACATATGCATCTTCCCTCAACAATTAAAAAATGAGGCATTGGTTTCTATATCCT
ms-3.seq    TACATTACATATGCATCTTCCCTCAACAATTAAAAAATGAGGCATTGGTTTCTATATCCT
            ************************************************************
```
