# Supplementary material for: Fine mapping of a male sterility gene ms-3 in a novel cucumber (Cucumis sativus L.) mutant
Source: Theor Appl Genet. 2017 Nov 13;131(2):449–60. doi: 10.1007/s00122-017-3013-2 (PMC5787221; doi:10.1007/s00122-017-3013-2)
Supplement: Supplementary file 3 — Supplementary material 3 (PDF 68 kb) [file 122_2017_3013_MOESM3_ESM.pdf]

Fig S3. Multiple sequence alignment of the non-synonymous single nucleotide polymorphism (SNP) loci of *Csa3M006660* from 8 Southern Chinese fertile lines (F1-F8), 10 Northern Chinese fertile lines(F9-F18), 8 Beit apha fertile lines (F19-F26), 2 Japanese fertile lines(F27-F28), WT and *ms-3.* (Black= Conversed region)

F1. seq	GTGAATCCTAAAACACGAATTTTAGAGTACACGATTCATGAACTTAGAAATGGCATTCA
F2. seq	GTGAATCCTAAAACACGAATTTTAGAGTACACGATTCATGAACTTAGAAATGGCATTCA
F3. seq	GTGAATCCTAAAACACGAATTTTAGAGTACACGATTCATGAACTTAGAAATGGCATTCA
F4. seq	GTGAATCCTAAAACACGAATTTTAGAGTACACGATTCATGAACTTAGAAATGGCATTCA
F5. seq	GTGAATCCTAAAACACGAATTTTAGAGTACACGATTCATGAACTTAGAAATGGCATTCA
F6. seq	GTGAATCCTAAAACACGAATTTTAGAGTACACGATTCATGAACTTAGAAATGGCATTCA
F7. seq	GTGAATCCTAAAACACGAATTTTAGAGTACACGATTCATGAACTTAGAAATGGCATTCA
F8. seq	GTGAATCCTAAAACACGAATTTTAGAGTACACGATTCATGAACTTAGAAATGGCATTCA
F9. seq	GTGAATCCTAAAACACGAATTTTAGAGTACACGATTCATGAACTTAGAAATGGCATTCA
F10. seq	GTGAATCCTAAAACACGAATTTTAGAGTACACGATTCATGAACTTAGAAATGGCATTCA
F11. seq	GTGAATCCTAAAACACGAATTTTAGAGTACACGATTCATGAACTTAGAAATGGCATTCA
F12. seq	GTGAATCCTAAAACACGAATTTTAGAGTACACGATTCATGAACTTAGAAATGGCATTCA
F13. seq	GTGAATCCTAAAACACGAATTTTAGAGTACACGATTCATGAACTTAGAAATGGCATTCA
F14. seq	GTGAATCCTAAAACACGAATTTTAGAGTACACGATTCATGAACTTAGAAATGGCATTCA
F15. seq	GTGAATCCTAAAACACGAATTTTAGAGTACACGATTCATGAACTTAGAAATGGCATTCA
F16. seq	GTGAATCCTAAAACACGAATTTTAGAGTACACGATTCATGAACTTAGAAATGGCATTCA
F17. seq	GTGAATCCTAAAACACGAATTTTAGAGTACACGATTCATGAACTTAGAAATGGCATTCA
F18. seq	GTGAATCCTAAAACACGAATTTTAGAGTACACGATTCATGAACTTAGAAATGGCATTCA
F19. seq	GTGAATCCTAAAACACGAATTTTAGAGTACACGATTCATGAACTTAGAAATGGCATTCA
F20. seq	GTGAATCCTAAAACACGAATTTTAGAGTACACGATTCATGAACTTAGAAATGGCATTCA
F21. seq	GTGAATCCTAAAACACGAATTTTAGAGTACACGATTCATGAACTTAGAAATGGCATTCA
F22. seq	GTGAATCCTAAAACACGAATTTTAGAGTACACGATTCATGAACTTAGAAATGGCATTCA
F23. seq	GTGAATCCTAAAACACGAATTTTAGAGTACACGATTCATGAACTTAGAAATGGCATTCA
F24. seq	GTGAATCCTAAAACACGAATTTTAGAGTACACGATTCATGAACTTAGAAATGGCATTCA
F25. seq	GTGAATCCTAAAACACGAATTTTAGAGTACACGATTCATGAACTTAGAAATGGCATTCA
F26. seq	GTGAATCCTAAAACACGAATTTTAGAGTACACGATTCATGAACTTAGAAATGGCATTCA
F27. seq	GTGAATCCTAAAACACGAATTTTAGAGTACACGATTCATGAACTTAGAAATGGCATTCA
F28. seq	GTGAATCCTAAAACACGAATTTTAGAGTACACGATTCATGAACTTAGAAATGGCATTCA
WT. seq	GTGAATCCTAAAACACGAATTTTAGAGTACACGATTCATGAACTTAGAAATGGCATTCA
ms-3. seq	GTGAATCCTAAAACACGAATTTTAGAGGACACGATTCATGAACTTAGAAATGGCATTCA
	*************************** *******************************
