# Supplementary material for: Fine mapping of a male sterility gene ms-3 in a novel cucumber (Cucumis sativus L.) mutant
Source: Theor Appl Genet. 2017 Nov 13;131(2):449–60. doi: 10.1007/s00122-017-3013-2 (PMC5787221; doi:10.1007/s00122-017-3013-2)
Supplement: Supplementary file 4 — Supplementary material 4 (PDF 105 kb) [file 122_2017_3013_MOESM4_ESM.pdf]

Fig S4. Alignment of protein sequences of cucumber Csa3M006660 and the closest homologs from 25 other plants species using CLUSTAL 2.1 The tyrosine residue created by the non-synonymous single nucleotide polymorphism (SNP) locus is shown in solid red boxes and the PHD domain is indicated with an empty red boxes.

```
XP_011652709.1[Cucumis_sativus]            ------MSISILESCKK-RKRRPKLFGFQTFGDPG-SPINPTG--PFRENIRIFLQQCAE
XP_007216520.1[Prunus_persica]             ------MSIPILEACKK-RKRRPKIYGFSSFCDPG-CPIGPNG--PFRDNIRLFLQECAE
XP_008244388.1[Prunus_mume]                ------MSIPILEACKK-RKRRPKIYGFSSFCDPG-FPIGPNG--PFRDNIRLFLQECAE
XP_018505942.1[Pyrus_x_bretschneideri]     ------MSIPILEACKK-KKRRPKIFGLQTFCDPG-CPISLIG--PFRDNVRVFLQECAE
OAY60550.1[Manihot_esculenta]              ------MSLPVLETCRK-RKRRPKVYSFHSFGDPG-CPMNPTG--PFRDNIRLFLQECAE
XP_008381356.1[Malus_domestica]            ------MSIPILEACKK-KKRRPKIFGLQSFCDPG-CPISLIG--PFRDNIRFFLQECAQ
XP_018816590.1[Juglans_regia]              ------MSISILEACKK-RKRRPKLYGFQNFGDPG-CPIDPKG--AFRDNIRLFLQECAD
XP_002263205.1[Vitis_vinifera]             ------MSIPILEACKK-RKRRPKLFGFHTFADPG-CPINPTS--HFCDNIRIFLRECAE
XP_004288593.1[Fragaria_vesca_subsp._vesca] ------MMIPTLEASKK-RKRRPKVYGFHSFGEPG-YPILPLG--PFRDNIRLFLQQCAE
XP_017981172.1[Theobroma_cacao]            ------MSIPIIETCRK-RKRRPKLFGLRSFAEPG-CPINPTG--PFRDNIRFFLKQCAE
XP_006372523.1[Populus_trichocarpa]        ------MSIPNLENCKK-RKRWPKLYDFNTFCEPD-CPINPRG--PFRDNMRLFLQQCAE
XP_011038253.1[Populus_euphratica]         ------MSIPNLENCKK-RKRWPKLYDFNTFCEPD-CPINPRG--PFRDNMRLFLQQCAE
XP_012086460.1[Jatropha_curcas]            ------MSLPFLEACRK-RKRRPKLYNFHSFGDPG-CPISPMG--PFRDNIRIFLQECAV
XP_006494662.1[Citrus_sinensis]            ------MSISTLESCKK-RKRRPKLFGFHTFMESG-CPISPTG--AFRDNVRQFLSACGE
XP_006421081.1[Citrus_clementina]          ------MSISTLESCKK-RKRRPKLFGFHTFMESG-CPISPTG--AFRDNVRQFLSACGE
XP_002533478.1[Ricinus_communis]           ------MSIPVLQTCKMMRKRKTKVYNFHSFGDPG-SAINPTG--PFRDNIRAFLQECAE
XP_010108961.1[Morus_notabilis]            ------MSIPIIEACKK-RKRRPKLFAFNAFGDES-GIPRFRC--PFRDKVRIFLEECAE
XP_003616245.2[Medicago_truncatula]        ------MSFSIIDASKK-RKRWTKIFPLQSFADPG-CPISPSG--PFRENVRLFLQEAGE
OAP18009.1[Arabidopsis_thaliana]           ------MPVPIIETCRK-RKRKPKVYNLQRFGEDG-FPIQRNG--AFRDQIRVFLRDCAE
BAK05033.1[Hordeum_vulgare_subsp._vulgare] MAAK---MVISLGSSRR-RKRGEVLFRFDSFCQPG-YPAQLAG--AFRDNVRTLLGLAHL
CDM85226.1[Triticum_aestivum]              ---------MVVNGRPLKKARTRAEARDFAGFPAAT--DGGAVG--TFREAVRGFLARHAR
XP_008657002.1[Zea_mays]                   ---------MVVNGRPLKRARARVEARDFARFPAAG--DCGAAG--TFREAVRGFLAKHAR
EAZ44935.1[Oryza_sativa_Japonica_Group]    MAPK---MVISLGSSRR-RKRGEMLFRFEAFCQPG-YPANFAGAGGFRDNVRTLLGFAHL
XP_002460302.1[Sorghum_bicolor]            MAAANKTMVISLGSSRR-RKRGEMLFRFESFCQPG-YPAPLAGGGAFRDNVRALLGLAHL
XP_003544360.1[Glycine_max]                ------MSFALIEACKK-RKRLPKFFRFNSFGDPGVVPIAPSG--PFRDNVRVFLQNAGE
```

```
XP_003578202.1[Brachypodium_distachyon]     MAAK---MVINLGSSRR-RKRGEVLFRFESFCQPG-YPAQLAG--PFRDNVRTLLGLAHL
                                                                          *               *    *  *

XP_011652709.1[Cucumis_sativus]             IEDY----------RIQEMPIWCTLLVHENKS-------FVVPLYTIEEDVKLSP-KPYC
XP_007216520.1[Prunus_persica]              LEDY----------SVQGMPTWCTLLVHDNRS-------LVVPLYIIEEDVKTSERP-FC
XP_008244388.1[Prunus_mume]                 LEDY----------SVQGMPTWCTLLVHDNRS-------LVVPLYTIEEDVKTSERP-FC
XP_018505942.1[Pyrus_x_bretschneideri]      REDY----------TVRGMPIWCTLLVHDNRS-------LVVPLYTIEEDVKTSEEP-FC
OAY60550.1[Manihot_esculenta]               PEDY----------NVEGMPIWCTLLVIESNN-------FVVPLYTIEENVHFSPNP-FC
XP_008381356.1[Malus_domestica]             REDY----------TVRGMPIWCTLLVHDNRS-------LVVPLYTIEEDVKTSEEP-FC
XP_018816590.1[Juglans_regia]               LHEN----------DIHAMPTWCTLLVHETRK-------FVVPLYTFEEDVKCSPRPPYC
XP_002263205.1[Vitis_vinifera]              LEDY----------NVDGMPTWCTLLVNENRG-------FVVPVYTIEESVKYAAKP-FC
XP_004288593.1[Fragaria_vesca_subsp._vesca] LESY----------SLLGMPVWCTLLVHDDRS-------LVLPLYTIEENVKDSDNP-FC
XP_017981172.1[Theobroma_cacao]             PEDY----------CVNGMPIWCTLLVHENKS-------SVVPLYTIKEDVKLSSNP-FC
XP_006372523.1[Populus_trichocarpa]         PEDY----------KVEGMSIWCTLLVIESKN-------FVVPLYTIEEDVKESVRP-FC
XP_011038253.1[Populus_euphratica]          PEDY----------KVEGMSIWCTLLVIESKN-------FVVPLYTIEEDVKESVRP-FC
XP_012086460.1[Jatropha_curcas]             RQDY----------TIEGMPIWSTLLLIESNS-------FVVPLYTIEENVKYSSNP-FC
XP_006494662.1[Citrus_sinensis]             LEDY----------KVEGMSIWCTLLVHESSS-------IVFPLYTIEEHVRHSPQP-YC
XP_006421081.1[Citrus_clementina]           LEDY----------KVEGMSIWCTLLVHESSS-------IVFPLYTIEEHVKHSPQP-YC
XP_002533478.1[Ricinus_communis]            PEDY----------NVDGMPVWCTHLAIESKS-------TVLPLYTIEENANDSPNP-FC
XP_010108961.1[Morus_notabilis]             VEDY----------QVLGMPIWCTLLVHENRN-------LVIPLYTIEETVKDSPLKSYC
XP_003616245.2[Medicago_truncatula]         LEDY----------TVMGNPLWCTFLIHEKKN-------LMVPFYALEEEVYNSSHP-FC
OAP18009.1[Arabidopsis_thaliana]            IEDY----------DIRGMPVWCTLLSHETKS-------SLIPLYIVEENVKHSSEP-YC
BAK05033.1[Hordeum_vulgare_subsp._vulgare]  EAG-----------VQGETRCWSFQLELHRHPP------TVVRLFVVEEEVAASPHR-QC
CDM85226.1[Triticum_aestivum]               LLPLPSIFSPAAAAAPPHLLTWRVSLRVGEEGAEEDAGGCGVELNVVEEDVLRSR-SVYC
XP_008657002.1[Zea_mays]                    LLPLPSIFSPAAAAAPPHLLIWRVSLRVGEAGEEESGG--GVELNVVEEDVLRSR-SVYC
EAZ44935.1[Oryza_sativa_Japonica_Group]     EAG-----------VHGETKCWSFQLELHRHPP------TVVRLFVVEEEVAASPHR-QC
XP_002460302.1[Sorghum_bicolor]             EAGG----------AHGDTKCWSFQLELHRHPP------TVVRLFVVEEVVDASPQR-QC
XP_003544360.1[Glycine_max]                 LEGY----------TVSGNPLWCILLIHDNSN-------AMAPLYTIEEHVDHSSHP-FC
```

```
XP_003578202.1[Brachypodium_distachyon]      EAG-----------VQGETRCWSFQLELQRHPP------TVVRLFIVEEEVAASPRR-QC
                                                                  *   *                      *          *

XP_011652709.1[Cucumis_sativus]              DQCRCSGWSNHFVSKRKYHIVIPLDDRWNKRLDDGGFDLDD-------------------
XP_007216520.1[Prunus_persica]               DHCRCTGWSNHFVSKRKYHIIIPMDDEWHKPLEDGICDLP--------------------
XP_008244388.1[Prunus_mume]                  DHCRCTGWSNHFVSKRKYHIIIPMDDEWHKPLEDGICDLP--------------------
XP_018505942.1[Pyrus_x_bretschneideri]       DQCRCTGWSNHFVSKRKYHMIIPVEDEWHKPQDNGILNLP--------------------
OAY60550.1[Manihot_esculenta]                DHCRCTGWGNNLVSKRKYHVIIPIAGEWSKRLEEGALDLH--------------------
XP_008381356.1[Malus_domestica]              DQCRCTGWSNXFVSKRKYHMIIPVDDEWHKPLDNGILDLP--------------------
XP_018816590.1[Juglans_regia]                DHCRCAGWSNHFVSKRKYHLIIPADEDWNKPLDDDLIDLQ--------------------
XP_002263205.1[Vitis_vinifera]               DHCRCTGWSNHFVSKRRYHIIIPIDDEWNKPLDDGVFDLQ--------------------
XP_004288593.1[Fragaria_vesca_subsp._vesca]  DHCRCTGWSNHFVSKRKYHMIIPMNDDWNKPLDDDIFDLS--------------------
XP_017981172.1[Theobroma_cacao]              DHCRCTGWSNHFVSKRKYHVVIPNDRDWNKPLEDGVLNIH--------------------
XP_006372523.1[Populus_trichocarpa]          DLCRCNGWSHNSVSKRKYHMIIPVDSEWSQKLEDGVCDLQ--------------------
XP_011038253.1[Populus_euphratica]           DLCRCNGWSHNSVSKRKYHMIIPVDSEWSQKLEDGVCDLQ--------------------
XP_012086460.1[Jatropha_curcas]              DHCRCTGWGDNFVSKRRYHLIIPNDSEWNEQLEG-VFDLQ--------------------
XP_006494662.1[Citrus_sinensis]              DQCRCSGWGNHYMSKRKYHWIIPIDCDWNKPLEDGVFDLQ--------------------
XP_006421081.1[Citrus_clementina]            DQCRCSGWANHYMSKRKYHWIIPIDCDWNKPLEDGVFDLQ--------------------
XP_002533478.1[Ricinus_communis]             DHCRCTGWGDNFVTRRRYHVIIPIDEEWNKRLEEGVFDVH--------------------
XP_010108961.1[Morus_notabilis]              DHCRCTGWSNHYVTKRKYHFLIPSDSRWNKPLDESVFDV---------------------
XP_003616245.2[Medicago_truncatula]          DHCRCVGWSGHFVSKRRYHFIIPMDDGWHKPLNEEALDDQ--------------------
OAP18009.1[Arabidopsis_thaliana]             DHCRCTGWSNHFVSKRKYHFIIPIDTEWSLPLEDDAFDSQ--------------------
BAK05033.1[Hordeum_vulgare_subsp._vulgare]   HLCRVIGWGRHLICSKRFHFVLP-KRESSVETDGLCYGIG------GADKAS--------
CDM85226.1[Triticum_aestivum]                DQCRVVGWSGHPVCGKRYHFIIENDSIQMAGRRRTCCLRCGTPMAAAESRCLLCNFDMEG
XP_008657002.1[Zea_mays]                     DQCRVVGWSGHPVCVKRYHFIIENDSSHLSGRRRTCCLRCGTPMAAGESRCALCNFDMDG
EAZ44935.1[Oryza_sativa_Japonica_Group]      HLCRHIGWGRHLICSKRYHFLLP-RRESAAEADGLCFAINHGGGG-GAEKASSK------
XP_002460302.1[Sorghum_bicolor]              LLCRHVGWGRHLICTKRFHFVLP-KRELSVEADGLHYGINHG-----PEKPS-K------
XP_003544360.1[Glycine_max]                  DHCRCVGWSGHFVPKRRYHFIIPMDNGWHKPLDEDSIDNQ--------------------
```

```
XP_003578202.1[Brachypodium_distachyon]      HLCRLIGWGRHLICSKRFHFLLP-KRESTVETDGLCYGISSSHGGGGTEKASSK------
                                               **  **          *

XP_011652709.1[Cucumis_sativus]              -----Q--------THLLHGLIHCNGFGHLLCVNGIEGGSKFLCGREVMDLWDRICTNLR
XP_007216520.1[Prunus_persica]               ---------------THLLHGLIHCNGYAHLVCVNGLEGGSKHLCGREIMDLWDRVCTNLR
XP_008244388.1[Prunus_mume]                  ---------------THLLHGLIHCNGYAHLVCVNGLEGGSKHLCGREIMDLWDRVCTNLR
XP_018505942.1[Pyrus_x_bretschneideri]       ---------------THLLHGLIHCNGFAHLVCINGLEGGSKHLYGREIMDLWDRICTNLR
OAY60550.1[Manihot_esculenta]                ---------------THILHGMIHCNGFGHLLCINGIEGGSKFLCGREIMDLWDRLCANLR
XP_008381356.1[Malus_domestica]              ---------------THLLHGLIHCNGFAHLVCINGLEGGSKHLYGREIMDLWDRICTNLR
XP_018816590.1[Juglans_regia]                ---------------NHLLHGLIHCNGFGHLICINGIEGGSKYLCGREIMDLWDRICTNLG
XP_002263205.1[Vitis_vinifera]               ---------------THLLHGMIHCNGFGHLLCINGIEGGSGYLCGREIMDLWDRICTILR
XP_004288593.1[Fragaria_vesca_subsp._vesca] ---------------THLLHGLIHCNGFAHLLCINGHEGGSKHLCGRELMDLWDRICLSLR
XP_017981172.1[Theobroma_cacao]              ---------------SHLLHGLIHCNGFGHLLCINGIEGGSKYLCGREIMDLWDRICEILR
XP_006372523.1[Populus_trichocarpa]          ---------------THLLHGLIHCNGFGHLLCINGREGGSKYLCGREIMDLWDRICASLR
XP_011038253.1[Populus_euphratica]           ---------------THLLHGLIHCNGFGHLLCINGREGGSKYLCGREIMDLWDRICASLR
XP_012086460.1[Jatropha_curcas]              ---------------NHLLHGLIHCNGFGHLLCINGIEGGSKILCGREIMDLWDRLCANLQ
XP_006494662.1[Citrus_sinensis]              ---------------THILHGLIHCNGFGHLLSINGIEGGSGYLCGREIMDLWDRICTNLR
XP_006421081.1[Citrus_clementina]            ---------------THILHGLIHCNGFGHLLSINGIEGGSGYLCGREIMDLWDRICTNLR
XP_002533478.1[Ricinus_communis]             ---------------THILHGLIHCNGFGHLLCINGIEGGSKTLSGREIMDLWDRLCINLR
XP_010108961.1[Morus_notabilis]              -----Q--------SHLLYGLIHGNGFGHLLGINGMEGGSKYLFGREIMDLWDRICGKLT
XP_003616245.2[Medicago_truncatula]          ---------------SHLLHGVIHCNGYGHLVCLNGIEGGSKFLSGREIMDLWDRICTNLR
OAP18009.1[Arabidopsis_thaliana]             ---------------SHVLHGLIHCNGFGHLVCVNGMESGSKYLCGREIVDFWDRLCNSLG
BAK05033.1[Hordeum_vulgare_subsp._vulgare]   -------KGTATSRGHLLHGIVHLNGYGHLVGLHGFEGGSDFVSGHQIMDLWDRICSALH
CDM85226.1[Triticum_aestivum]                EELEECGYLHLDDSSHLLHAVVHANGYGHLLRVNGREGGSRHLTGRDIMSFWDRLCKVLH
XP_008657002.1[Zea_mays]                     EEIEECAYLHLDDPTHLLHAVVHANGYGHLLRVNGREGGSRFLTGRDIMSLWDRLCKVLH
EAZ44935.1[Oryza_sativa_Japonica_Group]      -----GTTTTASSRGHLLHGVVHLNGYGHLVALHGLEGGSDFVSGHQIMDLWDRICSALH
XP_002460302.1[Sorghum_bicolor]              -----G---TATSRGHLLHGVVHLNGFGHLVALHGFEGGSEFVAGHQIMDLWDRICSSLN
XP_003544360.1[Glycine_max]                  ---------------KHLLHGVIHCNGYGHLLCVNGIEGGSKILSGREIIDLWDRICTNLR
```

```
XP_003578202.1[Brachypodium_distachyon]      -----GGTGTASSRGHLLHGVVHLNGYGHLVGLHGFEGGSDFVSGHQIMDLWDRICSALH
                                                            * *    * **  **    * * **    *      *** *  *

XP_011652709.1[Cucumis_sativus]              TRKITVEDLSKKRSMDLRLLHGVAYGHPWFGRWGYRFCRGSFGVKEHHYSRALEILSSLE
XP_007216520.1[Prunus_persica]               TRKITVEDVSKKRSMDLRLLHGIAYGHSWFGRWGYRFSHGSFGVTEHNYERALEILSSLE
XP_008244388.1[Prunus_mume]                  TRKITVEDVSKKRSMDLRLLHGIAYGHSWFGRWGYRFSHGSFGVTEHNYERALEILSSLE
XP_018505942.1[Pyrus_x_bretschneideri]       TRKISVEDASKKRSMDLRLLHGVAYGHSWFGRWGYRFCHGSFGVTAHNYERAIEILSSLA
OAY60550.1[Manihot_esculenta]                ARKVSVEDVSKKRSMDLRLLYGVAYGHPWYGRWGYKFCRGSFGVTKHNYNRAIEILSSLE
XP_008381356.1[Malus_domestica]              TRKISVEDASKKRSMDLRLLHGVAYGHSWFGRWGYRFCHGSFGVTAHKYERAIEILSSLA
XP_018816590.1[Juglans_regia]                TRKITVEDVSKKRSMDLRLLYGVAYGHSWFGRWGYRFCRGSFGVAEHNYDRAIEVLSSLE
XP_002263205.1[Vitis_vinifera]               TRKITVEDSSKKRFMDLRLLHGVAYGHPWFGRWGYRFCHGSFGVKEPNYERAIEILSSLE
XP_004288593.1[Fragaria_vesca_subsp._vesca]  TRKITVEDASKKRSMELRLLHGVAYGHPWFGRWGYKFCHGSFGVKEHIYNRALEILSSLE
XP_017981172.1[Theobroma_cacao]              ARKISVEDVSKKHGMDLRLLHGVAYGHTWFGRWGYKFCRGSYGVSEKNYGRAIEILSSSE
XP_006372523.1[Populus_trichocarpa]          TRKITVEDVSKKRSMDLRLLYGIAYGHPWFGRWGYKFCHGSFGVTEPIYFKAIEILSSME
XP_011038253.1[Populus_euphratica]           TRKITVEDVSKKRSMDLRLLYGIAYGHPWFGRWGYKFCHGSFGVTEPIYFRAIEILSTME
XP_012086460.1[Jatropha_curcas]              ARKISVQDVSKKRGMELRLLYGVAYGHSWFGRWGYKFCRGSFGVKLQNYNQAIEILCSLE
XP_006494662.1[Citrus_sinensis]              TCKVTVEDVSKKKSMDLRLLHGVAYGHSWFGRWGYKFFHGSFGVREQNYNRAIEILSSLE
XP_006421081.1[Citrus_clementina]            TCKVTVEDVSKKKSMDLRLLHGVAYGHSWFGRWGYKFFHGSFGVREQNYNRAIEILSSLE
XP_002533478.1[Ricinus_communis]             ARKISVEDVSKKRFMDLRLLYGVAYGHSWFGRWGYKFCRGSFGVTEHNYNSAIEILSSLA
XP_010108961.1[Morus_notabilis]              ARKITVADASKKRSMELRLLHGVAYGHPWFGRWGYGFCHGSFGVKDFNYKRALEILCSLE
XP_003616245.2[Medicago_truncatula]          ARYIAVEDASRKRSMDLRLLHGVAYGHSWFGRWGYGFCRGSFGVTQQNYDEAIEILGSLV
OAP18009.1[Arabidopsis_thaliana]             ARMITVEDLAKKRSVELRLLYGVAYGHSWFGRWGYKFCCGSFGVTKNEYENAIEALGSLE
BAK05033.1[Hordeum_vulgare_subsp._vulgare]   VRRVSLVDTARKGHMVLRLLHGVAYGDTWFGRWGYRYGRPSYGVALQSYQQSLHALQSIP
CDM85226.1[Triticum_aestivum]                VRKVTVMDISKKQGMDYRLLHAITTGHPWYGEWGYKFGAGSFAHTSDTYQEAVNVLSGIH
XP_008657002.1[Zea_mays]                     VRKVTVMDISKKHGMDYRLLHAVISGHPWYGQWGYKFGAGSFALTSDTYRNAVDMLSSIN
EAZ44935.1[Oryza_sativa_Japonica_Group]      VRTVSLVDTARKGHMELRLLHGVAYGETWFGRWGYRYGRPSYGVALPSYRQSLHVLGSMP
XP_002460302.1[Sorghum_bicolor]              VRKVSLVDTARKGHMELRLLHGVAYGDTWFGRWGYRFGRPSYGVALPSYQQSLHALQSVP
XP_003544360.1[Glycine_max]                  ARKIAVEDVSCKRSMDLRLLHGVAYGHSWFGRWGYRFCRGSSGVREREYNEAMTMLGSLG
```

```
XP_003578202.1[Brachypodium_distachyon]      VRKVSLVDTARKGHMVLRLLHGVAYGDTWFGRWGYRYGRPSYGVTLQSYQQSLHALQSVP
                                                    *   *     ***     *  * * ***     *       *      *

XP_011652709.1[Cucumis_sativus]              LDKIMHEVDYSDRGREVKQIIRHYRNLSETQLITLKDLLKFMLTVKY-VSAIEKKTVQPI
XP_007216520.1[Prunus_persica]               LERIIQAFSDMDQCDELKQIIRYYKNLSETQLITIKDLLRFMLTVKA-SVPAQKKSLMAT
XP_008244388.1[Prunus_mume]                  LERIIQAFSDMDQCDELKQIIRYYKNLSETQLITIKDLLRFMLTVKA-SVPAQKKSLMAT
XP_018505942.1[Pyrus_x_bretschneideri]       LERILQDFIDMDGCEELKQIIRYYRNLSETKLVTIKDLLRFMLTVKS-SVPGQKKSLMXX
OAY60550.1[Manihot_esculenta]                LDKIIQDFKNSDQCKEMKQIIHYYRDLSETLLMTFKDLLRFMLTVKS-CPCAQRKRSMAP
XP_008381356.1[Malus_domestica]              LERILQDFIDMDXCEELKQIIRYYRNLSETKLVTIKDLLRFMLTVKS-SVPGQKKSLMGP
XP_018816590.1[Juglans_regia]                LDKTIQDFSTMEKRREIKQLILHYRYMSETQLVTIKDLLRFMLTIKS-RAPLQINKPAAA
XP_002263205.1[Vitis_vinifera]               LDQIIEDFGCTDRCMKIRQIFRFYRDLSETQLITLKDILRIMLTLKS-RAPVQKKMNIVP
XP_004288593.1[Fragaria_vesca_subsp._vesca]  LEKIIQDFSDMDQYEELKKIVRYYRYLSETQLVTIKDLLRFMLTVKA-CFPAKRNSLLPT
XP_017981172.1[Theobroma_cacao]              LDKINQDFSDREQCRQIKCIIQHYRVLSESQLVTIRDLFKFMLTIKS-RSAVQKKSVTAI
XP_006372523.1[Populus_trichocarpa]          LEKIIQDFSDTSLSKSIKQIIHYYKDLSPTQLITFKDLLRFMLAIRS-CPCVWKKQSMTA
XP_011038253.1[Populus_euphratica]           LEKIIQDFSDTSLSKSIKQIIHYYKDLSPTQLITFKDFLRFMLAIRS-CPCVWKKQSMTA
XP_012086460.1[Jatropha_curcas]              LDKIIEEFKDTKVHQEMKQIISFYRNFSETPLITFKDLLRFMLIVKS-CPCAQKKRSIDS
XP_006494662.1[Citrus_sinensis]              LDNIIQDFCGTDLCREMKQIFHNYRDISETLLLTLKDLLRFMLTVKS-CASAQKKTIMTT
XP_006421081.1[Citrus_clementina]            LDNIIQDFCGTDLCREMKQIFHHYRDMSETLLLTLKDLLRFMLTVKS-CASAQKKTIMTT
XP_002533478.1[Ricinus_communis]             LNKIIQDFSNTNEYKEMKQMIDYYRNLSETQLITFRDLLRFMLTIKS-CPCAQKKLRLAA
XP_010108961.1[Morus_notabilis]              LDRIIEDFNETEQAKEIKQIIRLYRDLSETQLITLNELLRFMLTVKS-RAVGKPITTASI
XP_003616245.2[Medicago_truncatula]          LDDIVRDLSKTKYHKDVKQMIRFYRDMSETHIITIRELLRFMLTIKS-RRPVSKMTVTFS
OAP18009.1[Arabidopsis_thaliana]             IDQIEFDFGELRQSKEINQVFRYYREMSEGHLKTFRDLLRFMLIIKS-HASPQKLLPVTP
BAK05033.1[Hordeum_vulgare_subsp._vulgare]   LCVLVPHLS--CFSQELPLVVTKYQAISGHKLLNLGDLLRFMLELRT-------------
CDM85226.1[Triticum_aestivum]                LALYSSHRS--PIRTPLQNTIALYWSLSDRQLVTVRDLFRFIMHLLHQARKDAE------
XP_008657002.1[Zea_mays]                     LALYFSHRS--QIRTPLQNTIALYWALSNRQLVTLRDLFRFIMHLLHQGQK---------
EAZ44935.1[Oryza_sativa_Japonica_Group]      LCVLVPHLS--CFSQELPMVVTKYQAISGHKLLSLGDLLRFMLELRA-------------
XP_002460302.1[Sorghum_bicolor]              LCVLVPHLS--CFSQDLPVVVTKYQAISGHKLLNLGDLLRFMLELRT-------------
XP_003544360.1[Glycine_max]                  LDMIVKDLSETKYKTEIQQIIRCYRDMSETHIISLRDLLRFMLTVKSSRAPVPKITDTYS
```

```
XP_003578202.1[Brachypodium_distachyon]     LCVLVPHLAC-CFGQELPMVVTKYQAISGHKLLDLGDLLRFMLELRT-------------
                                                                   *   *

XP_011652709.1[Cucumis_sativus]             AKSP-----------------PPCRQSLQRNKQQSLVKE-KQIRYRKFATAISNMDSRWP
XP_007216520.1[Prunus_persica]              SAFSSS-------------TAKP--ATRAAHQIKPLMMKEKSVRYRKFTTVIAHMDSRWP
XP_008244388.1[Prunus_mume]                 SAFSSS-------------TAKP--ATRAALQIKPLMK-EKSVRYRKFNTVIAHMDSRWP
XP_018505942.1[Pyrus_x_bretschneideri]      AEVSSF-------------IVKS--ATKATLQVKPSMK-EKSAKCRKFSTILAHMDSRWP
OAY60550.1[Manihot_esculenta]               --TASP-------------SISKYLTRVAS-QKKPLMK-EKCIRYRKFSSLVGTLDSRWP
XP_008381356.1[Malus_domestica]             AEVSSF-------------IVKS--ATKATLQVKPSMK-EKSAKCRKFSTILAHMDSRWP
XP_018816590.1[Juglans_regia]               P---------------------PTSNSKPSIRNKPLVK-EKSLKYRSFTALAN-MDSRWP
XP_002263205.1[Vitis_vinifera]              DL--PA-------------SSTLKPSTRRSLQNRPPLK-DKTMKFKKFSTLITNMDSRWP
XP_004288593.1[Fragaria_vesca_subsp._vesca] AKPADV-------------MEKARPATRESLKIKPPMK-EKSSKYRKFSTVVSHMDSRWP
XP_017981172.1[Theobroma_cacao]             TA--PS-------------ASSQRNFIRISLPKKATSK-EKSSKCKRFTSVIAHMDSRWP
XP_006372523.1[Populus_trichocarpa]         T------------------TTSKPPINIVL-RRKPLIK-EKCMKYRNFSSLVGTMDSRWP
XP_011038253.1[Populus_euphratica]          T------------------TTSKPPINIVL-RRKPLIK-EKCMKYRNFSSLVGTMDSRWP
XP_012086460.1[Jatropha_curcas]             --------------------NVKSSMVIAS-QKKPVVK-EKYTRYRKFSSLVGTLDSRWS
XP_006494662.1[Citrus_sinensis]             TK--PS-------------K-------RMTLRNKSVVK-DKSVNYKRFSAVVAKMDSRWS
XP_006421081.1[Citrus_clementina]           TK--PS-------------K-------RMTLRIKSVVK-DKSVNYKRFSAVVAKMDSRWS
XP_002533478.1[Ricinus_communis]            DATASS-------------SAPKSTARVAALQKKPLVK-QKCTRYRKFSSLIGSLESRWP
XP_010108961.1[Morus_notabilis]             SSSSSSLFSS--------ISKPFTRTGLLHQSTKPFMKDSKPVKYKKFSTVIANMDSRWP
XP_003616245.2[Medicago_truncatula]         SPNAVSDTDSPSCSTSAFMSRNASKPVLSSSRSNFTMK-EKSARYKKFSTAVANMDSRWP
OAP18009.1[Arabidopsis_thaliana]            PLLTDS--------------PHQKRSSRLLLKKSDVADNDKSPKYRNYSTVAANLGSRWP
BAK05033.1[Hordeum_vulgare_subsp._vulgare]  ------------------------------------RLPATSVTAMDYRGIMSDASCRWS
CDM85226.1[Triticum_aestivum]               ---------------------------------------MSKPAMDEHREVESNVLCMWT
XP_008657002.1[Zea_mays]                    ---------------------------------------MSKPSADKHKELTSNELCAWT
EAZ44935.1[Oryza_sativa_Japonica_Group]     ------------------------------------RLPATSVTAMDYRGIMSEASCRWS
XP_002460302.1[Sorghum_bicolor]             ------------------------------------RLPATSVTAMDYRGIMSEASCRWS
XP_003544360.1[Glycine_max]                 ---AASDSTS-----SALTSRNSTKHTLPN-RSN-SMK-EKSVRYKKFSSAVTNMDSRWP
```

```
XP_003578202.1[Brachypodium_distachyon]     ------------------------------------RLPATSVTAMDYRGIMSDASCRWS
                                                                                                      *

XP_011652709.1[Cucumis_sativus]             ARRLEYAAEVIVKALEEKK-----SDKFSHGGNGMTRQDVRDAARLHIGDTGLLDYVLKS
XP_007216520.1[Prunus_persica]              QRRLEFAADVIVNALQEKK-----ERDFSHGG--MTRQDVRDTARLHIGDTGLLDYVLKS
XP_008244388.1[Prunus_mume]                 QRRLEFAADVIVNALQEKK-----ERDFSHGG--MTRQDVRDAARLHIGDTGLLDYVLKS
XP_018505942.1[Pyrus_x_bretschneideri]      TRRLEFAADVIVNALQEKK-----ES--GHGG--MTRQDVRDAARLHIGDTGLLDYVLKS
OAY60550.1[Manihot_esculenta]               TRRLEYAAEVIVNTLKEKK-----ADKLSKGG--MSRQDVRDAARMHIGDTGLLDYVLKS
XP_008381356.1[Malus_domestica]             TRRLEFAADVIVNTLQEKK-----ES--GHGG--MTRQDVRDAARLHIGDTGLLDYVLKS
XP_018816590.1[Juglans_regia]               ARRLEYAAEVIVNALKVKK-----EGKFCHGG--MTRQDLRDAARLHIGDTGLLDYVLKS
XP_002263205.1[Vitis_vinifera]              ARRLEYAADVIVNALREKRA----SECTH-DG--MTRQEVRDAARMHIGDTGLLDYVLKS
XP_004288593.1[Fragaria_vesca_subsp._vesca] AKRLEFAADVIVSALKEKK-----EKDFKNGG--MARQDVRDAARLHIGDTGLLDYVLKS
XP_017981172.1[Theobroma_cacao]             AKRLEYAAEVIVDALKEHKS----EFCH--GG--MTRQDLRDAARMHIGDTGLLDYVLKS
XP_006372523.1[Populus_trichocarpa]         TRRLQYAAEVIVDALKAKK-----EDKHSQEG--MTRQDVRDAARMHIGDTGLLDYVLKS
XP_011038253.1[Populus_euphratica]          TRRLQYAAEVIVDALKAKK-----EDKHSQEG--MTRQDVRDAARMHIGDTGLLDYVLKS
XP_012086460.1[Jatropha_curcas]             KKRLEYAAEVIVSALKEKR-----TDKFSQQG--MTRQDVRDAARMHIGDTGLLDYVLKS
XP_006494662.1[Citrus_sinensis]             ARRLESAAEVIVNALQEQKA----ENLGHGGG--MSRQKLRDAARMHIGDTGLLDYVLKS
XP_006421081.1[Citrus_clementina]           ARRLESAAEVIVNALQEQKA----ENLGRGGG--MSRQKLRDAARMHIGDTGLLDYVLKS
XP_002533478.1[Ricinus_communis]            ARRLEYTAGVIVDALKAKKK----ADKYSHGG--MTRQDVRDAARMHIGDTGLLDYVLKS
XP_010108961.1[Morus_notabilis]             PRRLEFAADVIVGALKEKK-----EDCFGNGG--MTRQDVRDAARLHIGDTGLLDYVLKS
XP_003616245.2[Medicago_truncatula]         TRRLEFAAQVIVEALKEDK-----AMKPGSSG--MTRQDVRDAARLHIGDTGLLDYVLKS
OAP18009.1[Arabidopsis_thaliana]            VRRLIFAAEVIVESLKEMK-------ALKQNG--MTRQDVRDSARLHIGDTGLLDYVLKS
BAK05033.1[Hordeum_vulgare_subsp._vulgare]   AKRVDMAARAVVDALRRSE-----AP----AARWVTRQEVRDAARTYIGDTGLLDFVLKS
CDM85226.1[Triticum_aestivum]               NEDINRAEAAMLKVLRAVQ-----------AGRWVSWRALRGAASKAVDSQELLDYSLRG
XP_008657002.1[Zea_mays]                    KEDLHRAEGAMLKVLQVVQ-----------TGQWVSWRALRGAASKAVDSQELLDYSLRG
EAZ44935.1[Oryza_sativa_Japonica_Group]     AKRVDMAARAVVDALRRAEP----------AARWVTRQEVRDAARAYIGDTGLLDFVLKS
XP_002460302.1[Sorghum_bicolor]             AKRVDMAARAVVDALRRTEP----------PARWVTRQEVRDAARAYIGDTGLLDFVLKS
XP_003544360.1[Glycine_max]                 TRRLEFAAQVIVDALKENK-----TVKLGSGG--MTRQDVRDAARLHIGDTGLLDYVLKS
```

```
XP_003578202.1[Brachypodium_distachyon]      AKRVDMAARAVVDALRRPASASDRAPGMPAAPRWVTRQEVRDAARAYIGDTGLLDFVLKS
                                                          *                         *  *        ***  *

XP_011652709.1[Cucumis_sativus]              LNNVIVGN-QIVRRAVNPKTRILEYTIHELR-----NGIQLTEEQ----ESTENSE----
XP_007216520.1[Prunus_persica]               LSNVIVGN-HIVCRAVNPTTRILEYTVHDLT---DGVKVSQPENE----VLPQSLP----
XP_008244388.1[Prunus_mume]                  LNNVIVGN-HIVCRAVNPTTRILEYTVHDLT---DGVKVSQPENE----ILPQSLP----
XP_018505942.1[Pyrus_x_bretschneideri]       MNNVIVGN-HIVCRAVNPATRILEYTVHDLA---DGVKVSEPGKE----IVAQSFQ----
OAY60550.1[Manihot_esculenta]                MNNVIVGT-HVVRRAVNPTTKILEYSIDELNGGVRPVRVTEPEAD----VVPEPLP----
XP_008381356.1[Malus_domestica]              MNNVIVGN-HIVCRAVNPATRILEYTVHDLA---DGVKVSEPGKE----IVAQSFQ----
XP_018816590.1[Juglans_regia]                MNNVVVGN-HIVCRTVNPTTRILEYSINELG----NEAISEPEIE----LLPKPLP----
XP_002263205.1[Vitis_vinifera]               MNNVIVGN-HVVCRAVNPATRVLEYTLKELG---KGTLVSELESE----MLPKPFP----
XP_004288593.1[Fragaria_vesca_subsp._vesca]  LNNVIVGN-QVVCRSVNPTTRILEYTVHDLD---DDDKVSEPEKE----ILPPPPQ----
XP_017981172.1[Theobroma_cacao]              MNNVIVGC-HIVRRAIN-TSRILEYTIDDID---NGFKAPEAELE----IHHKPLPDA--
XP_006372523.1[Populus_trichocarpa]          MNNVVVGK-YVVQRAVNPKTRILEYSIDEFGDGIIPVK-SEPES--------ETVP----
XP_011038253.1[Populus_euphratica]           MNNVVVGK-YVVQRAVNPKTRILEYSIEEFGDGIIPVK-SEPES--------ETVP----
XP_012086460.1[Jatropha_curcas]              MNNVIVGN-HVVHRAMNLETRILEYSIDELD--NRKVKVTEPEEE----EVADPIP----
XP_006494662.1[Citrus_sinensis]              MNNVIVGS-HIVHRAVNPATRLLEYTIHDLC---DGAGISEPGPE----ISDEPLP----
XP_006421081.1[Citrus_clementina]            MNNVIVGS-HIVHRAVNPATRLLEYTIHDLC---DGAGISEPGPE----ISDEPLP----
XP_002533478.1[Ricinus_communis]             MNNVIIGG-HVVRRAVNPKTKILEYSIDELGKENRSAGLTEIEA-----AVPEPLP----
XP_010108961.1[Morus_notabilis]              LNNVIVGN-QIIRRAVNPATRILEYTIHEVG-----EKVSEEQEL----VSNIPSP----
XP_003616245.2[Medicago_truncatula]          LNNVIVGN-YVVRRTVNSSSRILEYTIDELR---KGHQAPEMEHG-VLTVADKPQVESST
OAP18009.1[Arabidopsis_thaliana]             MNNVVVGD-VLVRRYVDPITRILHYTIQDLD---DAVKAKEPKKK-EAVVLEEITPLR--
BAK05033.1[Hordeum_vulgare_subsp._vulgare]   LGNHIVGN-YVVRRAMNPVTKVLEYCLEDVS----SVLPASVGAGAGVPAGHGKMRVR--
CDM85226.1[Triticum_aestivum]                LRGKLMDDGHFIAVRCNTETSAIEYRLETYS---NQSPVDATVFG-----PS--------
XP_008657002.1[Zea_mays]                     LGGKQLDNGLCVAVRCNAGTSAIEYRLESSS---VRSPANAATFG-----PS--------
EAZ44935.1[Oryza_sativa_Japonica_Group]      LGNHIVGN-YVVRRTMNPVTKVLEYCLEDVS----SVLPAVAAGGG--VPAQGKMRVR--
XP_002460302.1[Sorghum_bicolor]              LGNHIVGN-YVVRRAMNPVTKVLEYCLEDVS----SVLPAVGGGG-------GKMRVR--
XP_003544360.1[Glycine_max]                  LNNVIIGN-YVVRRMVNPSTRILEYTIHDLG---KGSKAPEENKPEVMAHADQQVVES--
```

```
XP_003578202.1[Brachypodium_distachyon]        LGNHIVGN-YVVRRAMNPVTKVLEYCLEDVS----SVLLSHG----------GKMRVR--
                                                                        *

XP_011652709.1[Cucumis_sativus]                ----PTVTPGKDIYNDVLCIYRSIFLDYP--------------ESEMVELATQGVLDSKH
XP_007216520.1[Prunus_persica]                 ---SASLVPGVDVYNDVLYLYEHVLLGYP--------------ESELVELATRAILDTKH
XP_008244388.1[Prunus_mume]                    ---SASLVPGVDVYNDVLYLYEHVLLGYP--------------ESELVELATRAILDTKH
XP_018505942.1[Pyrus_x_bretschneideri]         ---SATLFPGVDVYNDVLYLYEHVLLGYP--------------ESELVDLATRAVLDAKH
OAY60550.1[Manihot_esculenta]                  ---VLPLIPGADLYGDMAYLYTKVLLNYP--------------ESEVIELASQTVLDSKH
XP_008381356.1[Malus_domestica]                ---SATLFPGVDVYNDVLYLYEHVLLGYP--------------ESELVDLATRAVLDAKH
XP_018816590.1[Juglans_regia]                  ---ASTLVPGFDVHSDLVAVYKNLLLDYK--------------KLNLVELATQVVLDSKH
XP_002263205.1[Vitis_vinifera]                 ---EQSIVPGADVYNDVIYLYRNVLLNYP--------------DSELVELATRAVLDSKH
XP_004288593.1[Fragaria_vesca_subsp._vesca]    ---AAAPVSGIDVYSDVLYLYEHVLLGYP--------------ESGLVELATQAVLDSKH
XP_017981172.1[Theobroma_cacao]                ---LPALVPGTDVYDDVVYLYNNVLLDYP--------------ESEFLELATQAVLDSKH
XP_006372523.1[Populus_trichocarpa]            ---AQPLLPGADVNADVVFVYENVLFNYP--------------ESELVEVATQAILDSKH
XP_011038253.1[Populus_euphratica]             ---AQPLLPGADVNADVVFVYENVLFNYP--------------ESELVEVATQAILDSKH
XP_012086460.1[Jatropha_curcas]                ---VPSLVPGADLYGDMGYLYMKVLMNYP--------------ESELVETATQAILDSKH
XP_006494662.1[Citrus_sinensis]                ---PLALETGSDVYSEVVYLYMNVLLNYP--------------ESELVALATQAVLDSKH
XP_006421081.1[Citrus_clementina]              ---PLALETGSDVYSEVVYLYMNVLLNYP--------------ESELVALATQAVLDSKH
XP_002533478.1[Ricinus_communis]               ---AAP-VPGADLYADLGYLYLKVLLNYP--------------ESELAELATQTILDSKH
XP_010108961.1[Morus_notabilis]                ----ARIVPGVDVYNDVLFLYKHVLMEYP--------------DSEFVDLATQTILDTKH
XP_003616245.2[Medicago_truncatula]            LSSSSSSVPGNDVYSDVVYLYKNVLLGYP--------------DSEAVELAVQTILDCRH
OAP18009.1[Arabidopsis_thaliana]               --ILTPLKPGADVYGDLLLLYTNVLLNYP--------------ESELVRSATQAILDSKH
BAK05033.1[Hordeum_vulgare_subsp._vulgare]     -----FHLTRAQLMRDLVHLYRHVLKEP--------SQALTTGAFGAIPVAVRMILDIKH
CDM85226.1[Triticum_aestivum]                  ---VEHLAHDLRFLYDALLNPEIMLSSQP------------EVVGASAHNAAARILDCKQ
XP_008657002.1[Zea_mays]                       ---VDQLLHDLRFLYDALLNPESMLSSQP------------EVVGASAHSAAAKIIDCKQ
EAZ44935.1[Oryza_sativa_Japonica_Group]        -----FQLTRAQLMRDLVHLYRHVLKEP--------SQALTGGAFGAIPVAVRMVLDIKH
XP_002460302.1[Sorghum_bicolor]                -----FQLTRAQLMRDLTHLYRHVLREP--------SQALTTGAFGAIPVAARMVLDTKH
XP_003544360.1[Glycine_max]                    -----SWMLGNDVYSDALFLYKNVLLGYP--------------DSEAVDTAVQTILDSRY
```

```
XP_003578202.1[Brachypodium_distachyon]      -----FHLTRAQLMRDLVHLYRHVLKEPPTQLPLTASAGSGAAAFGAIPVAVRRVLDVKH
                                                                                                  *     *

XP_011652709.1[Cucumis_sativus]              FAKEWPLQDEE-EHLLT----FIIKLMPRLTFTHTDLELKSD-FMPSGEVVVLPLHTTIG
XP_007216520.1[Prunus_persica]               FVKECSFRDEE-EQLLT----FICQLLPSLMDMDIEFKRE----LPPGEIVVMPLHATIG
XP_008244388.1[Prunus_mume]                  FVKECSFRDEE-EQLLT----FICQLLPSSMDMDFEFKRE----LPPGEIVVMALHATIG
XP_018505942.1[Pyrus_x_bretschneideri]       FVKECPFRDDE-EQWLT----FFCQLLPSSTDKEIEFKRG----MPPGEVVVMPLHATIG
OAY60550.1[Manihot_esculenta]                FVKEWPFDDEE-DQLLR----FICQVMPNMIDLEANFNRE----LPPGEIVVLPLHATVA
XP_008381356.1[Malus_domestica]              FVKECPFRDDE-EQWLT----FFCQLLPSSTDKEIEFKRG----LPPGEVVVMPLHATIG
XP_018816590.1[Juglans_regia]                FVKEWPFTDEA-DQYLR----FICRLLPSFYDIETELNGK----FPPGEIVVVPLHATVG
XP_002263205.1[Vitis_vinifera]               FVKEWPFSDED-DQLLR----FVCHMMPSLSELE-IFTRE----LPPGEFIVVPPYATVG
XP_004288593.1[Fragaria_vesca_subsp._vesca]  FVKECSVRDDT-EKLLT----LFCQILPSFTDKEIDFKRD----LPPGEIVVMPLNATVG
XP_017981172.1[Theobroma_cacao]              FVKERPFRDED-DQLLR----FFCQVMPNLFGAENILTKK----SPAGELVTVPLHATVL
XP_006372523.1[Populus_trichocarpa]          FVKEWPFRVEN-DQLLS----FICQVMPTWNDLEAKFHRK----APPGEIIVLPLHASVL
XP_011038253.1[Populus_euphratica]           FVKEWPFRVEN-DQLLS----FICQVMPTWNDLEAKFHRK----APPGEIIVLPLHASVL
XP_012086460.1[Jatropha_curcas]              FVKEWPFENEE-DQLLR----YICQVMPNIIEAGTKFTRE----LPPGEIIMLPLHATVA
XP_006494662.1[Citrus_sinensis]              FVKEWPFRDED-DQFLR----FKCQVMPSFVDSETDLTAK----LPPGELVMIPLHSTVL
XP_006421081.1[Citrus_clementina]            FVKEWPFRDED-DQFLR----FKCQVMPSFVDSETDLTGK----LPPGELVMIPLHSTVL
XP_002533478.1[Ricinus_communis]             FVKVWPFKDEE-DELLR----FICQVMPNIIHLEVELKKE----LPPGEIVVLPLHSTVA
XP_010108961.1[Morus_notabilis]              LVKEWPFRDEE-DQLLT----FVCRLLPR-SFGDG-----------IAEIVRVPLHATIS
XP_003616245.2[Medicago_truncatula]          FVKEWKLRDEM-EQVLT----FICHLKPNFVENKSDLKGP-----SCGEIVTVPLHATVR
OAP18009.1[Arabidopsis_thaliana]             FIKEWPIWDNN-DTVLQ----FLCRINPSLVDVRSEQTTE----LPPGELVTVPLQATVY
BAK05033.1[Hordeum_vulgare_subsp._vulgare]   FVKDYHEGMTGTN------SGVVGHVYISLCCTLIVRNGSSE-LVPPYETVTVPAHATVG
CDM85226.1[Triticum_aestivum]                FIKHYDESAPESPPNP-----ILLAVRCSIELLDHPKDYT----APPVELVLLPATATLG
XP_008657002.1[Zea_mays]                     FIKHYDEHALRTPSNP-----FLLCVRCSIELLDHPKDYT----TPPEELVLLPASATLG
EAZ44935.1[Oryza_sativa_Japonica_Group]      FVKDYHEGQAAASS---NGGGGFGHPHINLCCTLLVSNGSPE-LAPPYETVTLPAHATVG
XP_002460302.1[Sorghum_bicolor]              FVKDYHEGFAPINS---VGAG---HVHMNLCCTLLVRNGSPELVAPPYETVTLPAHATVG
XP_003544360.1[Glycine_max]                  FVKEWPVRDEMKEQVLT----FICRLQPNFVDKKHELKGL-----ACGEVVVVPLHATVG
```

XP_003578202.1[Brachypodium_distachyon]	LVKDYHEAIMAAATANNSVGGIVGHVYVNMCCTLVVRDGSPE-LVPPYETVTVPAHATVG
	* *
XP_011652709.1[Cucumis_sativus]	EVKEAAEKALRDTYYVTEQFEVLAIENLENYE--DREVIFGAVESGAELFVKGMGIDLD-
XP_007216520.1[Prunus_persica]	ELKLAAETALRDTYCITERFVVKGIEGLDEME--DMEVLFGVVQSGAEVGVRGTGIDLD-
XP_008244388.1[Prunus_mume]	ELKLAAETALRDTYCITERFVVKGIEGLDEMD--DMEVLFGVVQSGAEVGVRGTGIDLD-
XP_018505942.1[Pyrus_x_bretschneideri]	ELKRAAESALRDTYCITEQFVVMGIEGLEEMD--DMEVLFGVAESGAEVGVRGSGIDLD-
OAY60550.1[Manihot_esculenta]	ELKQAATSALRDSYCILEKFVVTEIERMEELD--DWELLFGAVESGADLFMRGDGMDLN-
XP_008381356.1[Malus_domestica]	ELKRAAESALRDTYCITERFVVMGIEGLDEMD--DMEVLFGVAESGAEVGVRGSGIDLD-
XP_018816590.1[Juglans_regia]	ELKQAVENALRDTYCITEGFLVIEIKELEELE--DGEVLFGAVESGVELCVRGRGIDLE-
XP_002263205.1[Vitis_vinifera]	ELKETVERTLRDTYCIMEQVVVTEIEDMEGMT--DEEVLFGTIESGSEVWMRGTGMDLE-
XP_004288593.1[Fragaria_vesca_subsp._vesca]	ELKQEAENALRDTYCMTEQFVVTEIKGLEESD--DMDVLAGTVQSGTEVGVRGSGIDLD-
XP_017981172.1[Theobroma_cacao]	DLKQAVEKALRDTYCIMDKLVVTDIVDLGEMD--DKDVLFGALESGAKILVSGSGIDLD-
XP_006372523.1[Populus_trichocarpa]	ELKQEAESALRDTYCMLERFVVIEIEHMENLD--DKDLLCKFVESGAEIFVKGYGMDIN-
XP_011038253.1[Populus_euphratica]	ELKQEAESALRDTYCMLESFVVIEIEHMENLD--DKDLLCKFVKSGAEIFVKGYGMDIN-
XP_012086460.1[Jatropha_curcas]	ELKRAAENALKDTYYILENFVVTEIELMEELE--DWEVLFGVVESGSELFMKGNGVDLN-
XP_006494662.1[Citrus_sinensis]	ELKEAAESALRDTYCIMENLGVTDVVNMQKLD--DGELLFGAVESGSQVWIQGYGIDSD-
XP_006421081.1[Citrus_clementina]	ELKEAAESALRDTYCIMENLVVTDVGNMQKLD--DGELLFGAVESGSQVWIQGYGIDSD-
XP_002533478.1[Ricinus_communis]	ELKQAAENALRDTYCIMENFEVTEINQMEELM--DEELLFGSVESGAELFMRGNGMDLN-
XP_010108961.1[Morus_notabilis]	DLKEAVECALRDTYCVTEEIEVVEIDGLEEVE--DEEVLFGRVESGTEIRVRGVGVDTE-
XP_003616245.2[Medicago_truncatula]	DLKQTAEAALRDTYCIAERLIVTDIKELMDVE--DEEVIFGQIESGVKLSVRGIGIDLC-
OAP18009.1[Arabidopsis_thaliana]	DLKQAIEETFRDTYCILSNFVVTEIDEVEED--------MSLIGSCSALTVRGHGIDLE-
BAK05033.1[Hordeum_vulgare_subsp._vulgare]	ELKWEVQRLFRDMYLGLRTFTAECVVGIGAG--LDASPALGLIGVGSTVVVEGVVGEQQE
CDM85226.1[Triticum_aestivum]	ELKMLAARVFQETYLMFHSFQAEQLPEFPNLS--DTTPVKHVLGPSQLVRVRGRCTGDHR
XP_008657002.1[Zea_mays]	DLKVQASKAFQETYLMFQSFQTEQLPDFPNFS--DTTPVKHVLGSGQLLRVRGRCTGDYR
EAZ44935.1[Oryza_sativa_Japonica_Group]	ELKWEAQRVFSEMYLGLRSFAADSVVGVGAD--QEGLPVLGLVDVGSAVVVQGSVGEQIN
XP_002460302.1[Sorghum_bicolor]	ELKWEVQRLFREMYLGLRTFTAESVAGVGVS--KDACPVLGLIDVGSAVVIEGTVVEQQQ
XP_003544360.1[Glycine_max]	DLKRAAEAALRDTYCIAERLIVTDIKELMDVS--DEEVLFGLIQSGVELCVRGIAIDLL-

```
XP_003578202.1[Brachypodium_distachyon]      ELKWEVQRLFRDMYLALRTFTAESVVGIGIGPQQEASPVLGLIGVGSTVVVEGVVGSLQP
                                               *          *                                      *

XP_011652709.1[Cucumis_sativus]              ------------TPLKYQGGV---GTWKVRCECGTGDDDGERMVACDICEIWQHTRCCGID
XP_007216520.1[Prunus_persica]               ------------TPLRYEGGS---DTWMVRCECGARDDDGERMVACDICEVWQHTRCCGIE
XP_008244388.1[Prunus_mume]                  ------------TPLRYEGGS---DTWMVRCECGARDDDGERMVSCDICEVWQHTRCCGIE
XP_018505942.1[Pyrus_x_bretschneideri]       ------------TPLRYEGGS---DTWMVRCECGARDDDGERMVACDICEVWQHTRCCGIE
OAY60550.1[Manihot_esculenta]                ------------TELRYEGGP---DNWKVRCECGAQDDDGERMVACDICEVWQHTRCNGIE
XP_008381356.1[Malus_domestica]              ------------TPLRYEGGS---DTWMVRCECGARDDDGERMVACDICEVWQHTRCCGIE
XP_018816590.1[Juglans_regia]                ------------NRLRYQSGA---DTWMVRCECGARDDDGERMVACDLCEVWQHTRCCGIE
XP_002263205.1[Vitis_vinifera]               ------------AELKYEGGS---DNWRVSCECGALDDDGERMVACDICEVWQHTVCSGID
XP_004288593.1[Fragaria_vesca_subsp._vesca]  ------------TPLRYQGGS---DTWMVRCECGATDDDGERMVACDICEVWQHTRCCGME
XP_017981172.1[Theobroma_cacao]              ------------SNLRHEGGA---DNWIVRCECGAQDDDGERMVSCDICEVWQHTRCCGIE
XP_006372523.1[Populus_trichocarpa]          ------------SQLRYEGGS---DNWKVRCECGACDDDGERMVECDICEVWQHTRCNGID
XP_011038253.1[Populus_euphratica]           ------------SQLRYEGGS---DNWKVRCECGACDDDGERMVECDICEVWQHTRCNGID
XP_012086460.1[Jatropha_curcas]              ------------TELRYEAGP---DNWKVRCECGAQDDDGERMVACDICEVWEHTRCNGID
XP_006494662.1[Citrus_sinensis]              ------------SKLRYEGGN---DKWIVKCECGAQDDDGERMVACDICEVWQHTRCHGIE
XP_006421081.1[Citrus_clementina]            ------------SKLRYEGGN---DKWIVKCECGAQDDDGERMVACDICEVWQHTRCLGIE
XP_002533478.1[Ricinus_communis]             ------------TELRYESGP---DNWKVRCQCGAEDDDGERMVACDICEVWQHTRCNGIE
XP_010108961.1[Morus_notabilis]              ------------GPLMHQGGC---DPWKVVCECGARDDDGERMVACDICEVWQHTRCCGIE
XP_003616245.2[Medicago_truncatula]          ------------TPLKYQGGS---DNWKVRCECGAQDDDGERMVACDICEVWQHTRCCGID
OAP18009.1[Arabidopsis_thaliana]             ------------SKLKCQGGC---DTWMVKCICRARDDDGERMISCDVCEVWQHTRCCGID
BAK05033.1[Hordeum_vulgare_subsp._vulgare]   PAEEGD-QRKKAAAVCEGGGDV-GERVVDCVCGADDDDGERMACCDICEAWQHTRCAGVA
CDM85226.1[Triticum_aestivum]                ----------RIVQFRMERGL---ENWTVDCTCGAKDDDGERMMACDACGVWQHTRCSGIS
XP_008657002.1[Zea_mays]                     ----------RIVQFRMERGL---ENWTVDCSCGAKDDDGERMLACDICGVWQHTRCSGIS
EAZ44935.1[Oryza_sativa_Japonica_Group]      GEDHERKEEAAAAAVCEGSGG--GERVVDCACGAVDDDGERMACCDICEAWQHTRCAGIA
XP_002460302.1[Sorghum_bicolor]              LADEGVQPGNEAAAVSEGGGD--SERIVDCACGADDEDGERMACCDICEAWQHTRCAGIK
XP_003544360.1[Glycine_max]                  ------------TPLKYQGESESADNWKVRCECGAQDDDGERMVACDICEVWQHTRCYGID
```

```
XP_003578202.1[Brachypodium_distachyon]     AEEEEEESDEQRNGAVCEGSD---GERVVDCACGADDDDGERMACCDICEAWQHTRCAGVA
                                                                       * * *   * *****  ** *  * ** * *

XP_011652709.1[Cucumis_sativus]             DADNVPLLFVCAACCDSLGQL--KI-----------------------------------
XP_007216520.1[Prunus_persica]              DADTVPPLFVCSACCVSLVPP--KFEP-CDR-TLFCLSLSIFWTVRNGGSESLAFLENAT
XP_008244388.1[Prunus_mume]                 DADTVPPLFVCSACCVSLVPP--KFEP-CLR-FDCSDAFSIFCTH---------------
XP_018505942.1[Pyrus_x_bretschneideri]      DADTVPPLFVCSACCVSLVPP--RIEP-CLR-FDCSDAFSICCRH---------------
OAY60550.1[Manihot_esculenta]               DSGTVPPLFICAGCCDSLGHS--RGES-QEE-LENSDDLLMIP------ATEYVAQFLE-
XP_008381356.1[Malus_domestica]             DADTVPPLFVCSACCVSLVPP--RIEP-CLR-FDCSDAFSICCRH---------------
XP_018816590.1[Juglans_regia]               DTEAVPPLFVCSGCCVSLGTL--KTEA-SCA-FECSDTLLMST-------DTFGLELGY-
XP_002263205.1[Vitis_vinifera]              DSETVPPLFVCPKCCALLVPA--RSES-SD-----CGRVQLAP------VYQFGMELLY-
XP_004288593.1[Fragaria_vesca_subsp._vesca] DADEVPRLFICSTCYVSLVPP--KTEPAALR-FDCSTAFLEWRH----------------
XP_017981172.1[Theobroma_cacao]             DSEAVPPLFVCPGCCASLGPP--MSES-PLT-YQSSDDLLLDS------ETVYGMD-LEY
XP_006372523.1[Populus_trichocarpa]         DADTVPQLFICSGCCDSLLPG--KTET-HQR-FESSDDLLMIPAAIG-YGAEAAEPFYE-
XP_011038253.1[Populus_euphratica]          DADTVPQLFICSGCCDSLLPG--KTET-HQR-FESSDDLLMIPAAIG-YGAEAAEPFYQA
XP_012086460.1[Jatropha_curcas]             DSKTVPPLFICTECCNSVVKP--TNEL-QVE-FENSDHLLMVP------PNAYVAELVK-
XP_006494662.1[Citrus_sinensis]             DSGTVPPLFVCPRCCSSFAPS--RTES-SFR-FLGSDDLLLVP------ETEYGAERVVF
XP_006421081.1[Citrus_clementina]           DSGTVPPLFVCPRCCSSFAPS--RTES-SF------------------------------
XP_002533478.1[Ricinus_communis]            DSEAVPLLFVCTRCCDSMIKS--RKKV-KVE-IGNSEDLLMIPAIFQDFANDGIGMLL--
XP_010108961.1[Morus_notabilis]             DAETVPPLFVCSACCDSLVPL--RANHEPSP-DALSDDLLLLTP-----QVDYSLAIGY-
XP_003616245.2[Medicago_truncatula]         DSETVPPLFVCSGCCDSLMPP--RIESSSFGMVDCADSFLVSPE-----PTHLLEYYGY-
OAP18009.1[Arabidopsis_thaliana]            DSDTLPPLFVCSNCCEEFAEQQ-RKVLQPKYEFPSSENVFLLESADDFFGDQRCLGMIFP
BAK05033.1[Hordeum_vulgare_subsp._vulgare]  DTEDVPHVFLCSRCDNDVASFPALNC----------------------------------
CDM85226.1[Triticum_aestivum]               DFEEVPEKFICRKCASPRKGKGGRGGGGGSGGGRMEMASAGRCKDEIGSSVGGAGKIGRL
XP_008657002.1[Zea_mays]                    DFEEVPENFICRKCASPRKGDG-RGGGGSNGGGTMEVTASGRCKDEIGASVGGAGKYGRM
EAZ44935.1[Oryza_sativa_Japonica_Group]     DTEDAPHVFLCSRCDNDVVSFPSFNC----------------------------------
XP_002460302.1[Sorghum_bicolor]             DTDDAPHVFVCNRCDNDVLSFPPLSC----------------------------------
XP_003544360.1[Glycine_max]                 DSETVPPLFVCTGCCDSVLVPSSRTESAAFG-VDSVDSFLISED-----PTTLLLDYEYG
```

```
XP_003578202.1[Brachypodium_distachyon]      DADDVPHVFLCSRCDNDVLSFPSFNC----------------------------------
                                             *    *  * *  *

XP_011652709.1[Cucumis_sativus]              --------
XP_007216520.1[Prunus_persica]               T-------
XP_008244388.1[Prunus_mume]                  --------
XP_018505942.1[Pyrus_x_bretschneideri]       --------
OAY60550.1[Manihot_esculenta]                --------
XP_008381356.1[Malus_domestica]              --------
XP_018816590.1[Juglans_regia]                --------
XP_002263205.1[Vitis_vinifera]               --------
XP_004288593.1[Fragaria_vesca_subsp._vesca]  --------
XP_017981172.1[Theobroma_cacao]              DNCIRILP
XP_006372523.1[Populus_trichocarpa]          --------
XP_011038253.1[Populus_euphratica]           --------
XP_012086460.1[Jatropha_curcas]              --------
XP_006494662.1[Citrus_sinensis]              NDDIGMSL
XP_006421081.1[Citrus_clementina]            --------
XP_002533478.1[Ricinus_communis]             --------
XP_010108961.1[Morus_notabilis]              --------
XP_003616245.2[Medicago_truncatula]          --------
OAP18009.1[Arabidopsis_thaliana]             EENYLL--
BAK05033.1[Hordeum_vulgare_subsp._vulgare]   --------
CDM85226.1[Triticum_aestivum]                ATVG----
XP_008657002.1[Zea_mays]                     ATVG----
EAZ44935.1[Oryza_sativa_Japonica_Group]      --------
XP_002460302.1[Sorghum_bicolor]              --------
XP_003544360.1[Glycine_max]                  Y-------
```

XP_003578202.1[Brachypodium_distachyon]   ---------
