# Supplementary material for: Fine mapping of a male sterility gene ms-3 in a novel cucumber (Cucumis sativus L.) mutant
Source: Theor Appl Genet. 2017 Nov 13;131(2):449–60. doi: 10.1007/s00122-017-3013-2 (PMC5787221; doi:10.1007/s00122-017-3013-2)
Supplement: Supplementary file 5 — Supplementary material 5 (PDF 77 kb) [file 122_2017_3013_MOESM5_ESM.pdf]

Fig S5. Protein sequence alignment of cucumber Csa3M006660 and *Arabidopsis thaliana* MMD1. The sequences shared 48.96% identity.
(Black= Conversed region; Yellow highlight=PHD domain; Red highlight=SNP loci)

```
MMD1          MPVPIIETCRKRKRKPKVYNLQRFGEDGFPIQRNGAFRDQIRVFLRDCAEIEDYDIRGMTVWCTLLSHETKSSLIPLYIVEENVKHSSEPYCDHCRCTGWS
Csa3M006660   MSISILESCKKRKRRPKLFGFQTFGDPGSPINPTGPFRENIRIFLQQCAEIEDYRIQEMPIWCTLLVHENKSFVVPLYTIEEDVKLSPKPYCDQCRCSGWS
              *   * * * **** **    * **  * **   * **  ** **  ******* *  *  ***** ** **   ***  ** ** *  **** *** ***

MMD1          NHFVSKRKYHFIIPIDTEWSLPLEDDAFD--SQSHVLHGLIHCNGFGHLVCVNGMESGSKYLCGREIVDFWDRLCNSLGARMITVEDLAKKRSVELRLLYG
Csa3M006660   NHFVSKRKYHIVIPLDDRWNKRLDDGGFDLDDQTHLLHGLIHCNGFGHLLCVNGIEGGSKFLCGREVMDLWDRICTNLRTRKITVEDLSKKRSMDLRLLHG
              **********  ** *  *   * *  **   * * ************* **** * *** *****  * *** *  *  * ****** ****  **** *

MMD1          VAYGHSWFGRWGYKFCCGSFGVTKNEYENAIEALGSLEIDQIEFDFGELRQSKEINQVFRYYREMSEGHLKTFRDLLRFMLIIKSHASPQKLLPVTPPLLT
Csa3M006660   VAYGHPWFGRWGYRFCRGSFGVKEHHYSRALEILSSLELDKIMHEVDYSDRGREVKQIIRHYRNLSETQLITLKDLLKFMLTVK-YVSAIEKKTVQP--IA
              ***** ******* ** *****    *  * * * *** * *           *  *  * **  **  * *  *** ***  *   *      * *

MMD1          DSPHQKRSSRLLLKKSDVADNDKSPKYRNYSTVAANLGSRWPVRRLIFAAEVIVESLKEMKALKQ----NGMTRQDVRDSARLHIGDTGLLDYVLKSMNNV
Csa3M006660   KSPPPCRQS-LQRNKQQSLVKEKQIRYRKFATAISNMDSRWPARRLEYAAEVIVKALEEKKSDKFSHGGNGMTRQDVRDAARLHIGDTGLLDYVLKSLNNV
               **   * * *   *       *   **   *   *  **** ***  ******  * * *  *     ********* ***************** ***

MMD1          VVGDVLVRRYVDPITRILHYTIQDLDDAVKAKEPKKKEAVVLEEITPLRILTPLKPGADVYGDLLLLYTNVLLNYPESELVRSATQAILDSKHFIKEWPIW
Csa3M006660   IVGNQIVRRAVNPKTRILEYTIHELRNGIQLTE--EQESTENSEPT-------VTPGKDIYNDVLCIYRSIFLDYPESEMVELATQGVLDSKHFAKEWPLQ
               **   *** * * **** ***  *       *    *     * *         ** * * * *  *    * ***** *  ***  ****** ****

MMD1          DNNDTVLQFLCRINPSLVDVRSE---QTTELPPGELVTVPLQATVYDLKQAIEETFRDTYCILSNFVVTEIDEVE--EDMSLIG----SCSALTV
Csa3M006660   DEEEHLLTFIIKLMPRLTFTHTDLELKSDFMPSGEVVVLPLHTTIGEVKEAAEKALRDTYYVTEQFEVLAIENLENYEDREVIFGAVESGAELFV
              *     * *     * *            * ** *  **  *    * * *   ****     * *  *   *  **   *     *   * *

MMD1          RGHGIDLESKLKCQGGCDTWMVKCICRARDDDGERMISCDVCEVWQHTRCCGIDDSDTLPPLFVCSNCCEEFAEQQRKVLQPKYEFPSSENVFLLE
```

```
Csa3M006660      KGMGIDLDTPLKYQGGVGTWKVRCECGTGDDDGERMVACDICEIWQHTRCCGIDDADNVPLLFVCAACCDSLGQLK-------------------
                  * ****   ** ***  ** * * *   *******  ** ** *********** *  * ****  **

MMD1             SADDFFGDQRCLGMIFPEENYLL
Csa3M006660      ------------------------
```
