# Supplementary material for: Fine mapping of a male sterility gene ms-3 in a novel cucumber (Cucumis sativus L.) mutant
Source: Theor Appl Genet. 2017 Nov 13;131(2):449–60. doi: 10.1007/s00122-017-3013-2 (PMC5787221; doi:10.1007/s00122-017-3013-2)
Supplement: Supplementary file 6 — Supplementary material 6 (PDF 49 kb) [file 122_2017_3013_MOESM6_ESM.pdf]

Table S1. Gene primers designed for KASPar

Marker names	SNP position	Alle le X	Allele Y	Primer Allele X (5′-3′)<br>Primer Allele Y (5′-3′)	Primer Common (3′-5′)
C304430G	304430	C	G	AATTACATGAATAAGTGTTCGTAATTTCG<br>AATTACATGAATAAGTGTTCGTAATTTCC	AAACTTCAAGTTTAGGATAGAATCGGTTTGA
G564531C	564531	G	C	GGTTTGGAATCTTGCTTGGCATTG<br>GGTTTGGAATCTTGCTTGGCATTC	CAATCAACCATATTCAGTTTAATCAACTAAACAAA
A701466G	701466	A	G	ATCTAGAAACCAAATAAAAACTATAGCCAA<br>CTAGAAACCAAATAAAAACTATAGCCAG	AATTTAGTTTCAAAATTTGTTTATTAATAAAAATATACATCTCT
G729940C	729940	G	C	GGAACCCCTTCTGAAGCTGTG<br>GGAACCCCTTCTGAAGCTGTC	GTAGCTATAAGAAAGGGCAGAGACC
T785141C	785141	T	C	GGGTCACGCAGATGGGTATTGA<br>GGTCACGCAGATGGGTATTGG	TCCAATCACCACTTCCTACAATTTATCG
A806246C	806246	A	C	GCCATTTCTAAGTTCATGAATCGTGTA<br>CCATTTCTAAGTTCATGAATCGTGTC	TAGTTCGCCGTGCAGTGAATCCTAA
T861262G	861262	T	G	TTTCAAAATCATTTCAAACTTACTGATAACTA<br>TCAAAATCATTTCAAACTTACTGATAACTC	TAAATTAAGAGACTGAATTTATTACTTAACCAAATAC
T1031386G	1031386	T	G	TACGTGAATATTTTCTTTTTCTTTATACGTAT<br>CGTGAATATTTTCTTTTTCTTTATACGTAG	ATTTAGAAAACACACGTATCAAAATTAACTTAGC
T1101289C	1101289	T	C	AAGACTAATATGCCCTTCCTCTTCTA<br>GACTAATATGCCCTTCCTCTTCTG	ATTCCAAGTTGGGATTGAATGGAAATGG
T1508343G	1508343	T	G	TATGTACAGCATCAACAAGTGTGCA<br>TGTACAGCATCAACAAGTGTGCC	ATCATCTGTCTCCAATTCACTTGAAAACTTT
A2179014C	2179014	A	C	TTCGAACATATACAAAAGTAGATATATCAAAA<br>CGAACATATACAAAAGTAGATATATCAAAC	GTGATTCATTGAGAATATCTATTTAATTGTACTTG
