# Supplementary material for: Fine mapping of a male sterility gene ms-3 in a novel cucumber (Cucumis sativus L.) mutant
Source: Theor Appl Genet. 2017 Nov 13;131(2):449–60. doi: 10.1007/s00122-017-3013-2 (PMC5787221; doi:10.1007/s00122-017-3013-2)
Supplement: Supplementary file 7 — Supplementary material 7 (PDF 42 kb) [file 122_2017_3013_MOESM7_ESM.pdf]

Table S2. Putative genes in the 76 kb region of cucumber chromosome 3 predicted to contain *Ms-3*

Genes	Position	Predicted function
Csa3M006630	Chr3:782240-786910	COBW domain-containing protein 2-like
Csa3M006640	Chr3:787921-798914	DNA (cytosine-5)-methyltransferase CMT2-like
Csa3M006650	Chr3:802042-805053	PHD finger protein MALE MEIOCYTE DEATH 1-like
Csa3M006660	Chr3:805509-807682	PHD finger protein MALE MEIOCYTE DEATH 1-like
Csa3M006670	Chr3:814253-819247	uncharacterized LOC101207845
Csa3M006680	Chr3:820895-825452	protein LTV1 homolog
Csa3M006690	Chr3:826762-830720	phosphoribulokinase, chloroplastic-like
Csa3M006700	Chr3:831598-836257	secretory carrier-associated membrane protein 4-like
Csa3M006710	Chr3:838820-841281	uncharacterized LOC101208810
Csa3M006720	Chr3:844556-849288	26S proteasome non-ATPase regulatory subunit 13-like
Csa3M006730	Chr3:848589-855285	uncharacterized LOC101207353
